# Large-Scale Automatic Feature Selection for Biomarker Discovery in High-Dimensional OMICs Data

**DOI:** 10.3389/fgene.2019.00452

**Published:** 2019-05-16

**Authors:** Mickael Leclercq, Benjamin Vittrant, Marie Laure Martin-Magniette, Marie Pier Scott Boyer, Olivier Perin, Alain Bergeron, Yves Fradet, Arnaud Droit

**Affiliations:** ^1^Centre de Recherche du CHU de Québec–Université Laval, Québec City, QC, Canada; ^2^Département de Médecine Moléculaire, Université Laval, Québec City, QC, Canada; ^3^Institute of Plant Sciences Paris Saclay IPS2, CNRS, INRA, Université Paris-Sud, Université Evry, Université Paris-Saclay, Paris Diderot, Sorbonne Paris-Cité, Orsay, France; ^4^UMR MIA-Paris, AgroParisTech, INRA, Université Paris-Saclay, Paris, France; ^5^Digital Sciences Department, L'Oréal Advanced Research, Aulnay-sous-bois, France; ^6^Département de Chirurgie, Oncology Axis, Université Laval, Québec City, QC, Canada

**Keywords:** machine learning, omics, biomarkers signature, feature selection, precision medicine

## Abstract

The identification of biomarker signatures in omics molecular profiling is usually performed to predict outcomes in a precision medicine context, such as patient disease susceptibility, diagnosis, prognosis, and treatment response. To identify these signatures, we have developed a biomarker discovery tool, called BioDiscML. From a collection of samples and their associated characteristics, i.e., the biomarkers (e.g., gene expression, protein levels, clinico-pathological data), BioDiscML exploits various feature selection procedures to produce signatures associated to machine learning models that will predict efficiently a specified outcome. To this purpose, BioDiscML uses a large variety of machine learning algorithms to select the best combination of biomarkers for predicting categorical or continuous outcomes from highly unbalanced datasets. The software has been implemented to automate all machine learning steps, including data pre-processing, feature selection, model selection, and performance evaluation. BioDiscML is delivered as a stand-alone program and is available for download at https://github.com/mickaelleclercq/BioDiscML.

## Introduction

The identification of biomarkers that are indicative of a specific biological state is a major research topic in biomedical applications of computational biology (Liu et al., 2014; Beerenwinkel et al., 2016; Zhang et al., 2017). With the emergence of high-throughput molecular profiling technologies and their decreasing costs, traditional medicine is moving to precision medicine to improve disease diagnosis, and to propose tailored interventions to individuals. Research studies involving cohorts of patients aim to discover patterns that establish risk stratification and discriminate patient states, such as diseased vs. controls, disease type, etc. These last years, clinical and biology research turned toward extensive usage of OMICs (i.e., proteomics, transcriptomics, metabolomics, genomics, etc.) technologies, which include microarrays, mass spectrometry, and whole exome/genome and RNA sequencing. Specific patterns associated with a clinical outcome of interest (e.g., disease diagnostic, prognostic), called biomarker signatures, can be derived from these high-dimensional technologies outputs (e.g., gene expression, polymorphisms) (Lin et al., 2017). These signatures, which are measurable indicators for predicting a biological phenomenon, are usually identified using machine learning (Pasolli et al., 2016) or statistical multivariate analysis approaches (Rohart et al., 2017c).

Biomarker signature identification from disease-derived omics datasets is a challenging task involving many pitfalls. First, the datasets are generally highly unbalanced, where the features (e.g., genes, peptides, metabolites…), also called attributes or variables, largely outnumber the samples. In addition, patients are unequally distributed among measured outcomes. Second, the molecular profiles are often heterogeneous (e.g., sub-phenotypes in cancer data), of diverse types (e.g., categorical, continuous), and scattered over multiple inputs (Libbrecht and Noble, 2015). To identify sets of predictive biomarker signatures from omics data, a few non-commercial methods have been implemented in R packages (Lê Cao et al., 2009; Taverner et al., 2012; Cun and Fröhlich, 2014; Rohart et al., 2017b). These toolkits have adopted diverse multivariate projection-based methods including principal component analysis (Wold, 1975), independent component analysis (Yao et al., 2012), multi-group partial least squares regression (Eslami et al., 2013), canonical correlation analysis (Hotelling, 1936), K-means clustering (Hartigan and Wong, 1979), and associated visualizations. Recently, other research teams have proposed approaches in machine learning (ML) (Janevski et al., 2009; Cun and Fröhlich, 2013; Lagani et al., 2013; Swan et al., 2013, 2015; Butti et al., 2014; Kong et al., 2014; Kourou et al., 2015), a branch of artificial intelligence that holds a great potential for pattern recognition in complex diseases datasets. ML has already shown its ability to identify key features (markers) and modeling predictive biomarker signature in a variety of fields, including cancer research (Matsumura et al., 2010; Cima et al., 2011; Cui et al., 2011; Roth et al., 2011; Fröhlich and Cun, 2012; Kourou et al., 2015), neurology (Daoqiang and Dinggang, 2012; Deshpande et al., 2013; Fekete et al., 2013), immunology (Sutherland et al., 2011), skin diseases (Johansson et al., 2011), etc. However, all these techniques are complex to use and are out-of-reach for non-programmers and non-ML experts. Furthermore, the software implemented specifically for omics data are still rare and are strictly limited to specific ML algorithms for feature selection (also called “attribute selection”) or classification (Butti et al., 2014). Hence, there is an unmet need to develop user-friendly computational approaches for using machine learning in a biomedical context that are dedicated to biologists and clinical researchers. These approaches must be able to identify complex patterns and predict outcomes in various biological or clinical fields (e.g., disease diagnosis, prognosis, therapeutics), thus helping to understand the biology behind a measured outcome.

Considering the complexity of the ML approach, we present in this paper a software called BioDiscML (*Bio*marker *Disc*overy by *M*achine *L*earning), which aims to greatly facilitate the work required for biomarker signature identification from high-dimensional data, such as gene expression, by automating the ML approach. Some non-commercial automatic software already exists to facilitate the choice of learning algorithms and perform hyper-parameter optimization, such as Auto-weka (Thornton et al., 2013), auto-Sklearn (Feurer et al., 2015), autoML (Feurer et al., 2015), and preconfigured pipelines in Orange canvas (Demšar et al., 2013). But they are not explicitly designed to answer biological problems, lack of user-friendly experience for non-ML experts, some focusing only on hyperparameter optimization, and may be complex to parallelize to decrease calculation time. We aim here to fill the gap, providing BioDiscML the capacity to test large number of feature subsets and models in order to obtain the most performant signature to predict a measured outcome. BioDiscML uses an exhaustive search approach, which systematically enumerates a pre-defined set of possible candidates for a solution and test whether each candidate satisfies the problem statement. BioDiscML can also merge files from different sources, search for the most predictive combination of feature subsets and machine learning classifiers, train a model, evaluate predictive performances, parallelize the computation, and search for correlated features.

## Materials and Methods

BioDiscML is a tool that automates main ML steps by implementing methods for feature and model selection. In this section, we describe the program procedures separated in three main components: preprocessing, feature selection and model selection. We also present all supported models (see Supplementary Materials), evaluation metrics, feature search methods, best model selection and correlated features search approaches. Finally, we have summarized the real-life datasets we used to compare BioDiscML against various existing tools.

### BioDiscML Software

BioDiscML is a biomarker discovery software that supports classification (categorical class) and regression (numerical class) problems. It is written in JAVA 8 language (Fischer, 2015) and use Weka 3.8 machine learning library (Holmes et al., 1994; Hall et al., 2009; Witten et al., 2016). It automates several machine learning steps aiming to identify predictive models. To this purpose, BioDiscML can routinely perform data preprocessing, features dimension reduction, a combined feature and model selection strategy, identify best models, and search correlated features. All machine learning generated models are evaluated by various cross validation procedures. All steps are configured with editable default parameters. Advanced parameters can also be modified by the user. Some basic information is needed to start the program such as: input dataset(s), class label name, problem type (regression or classification).

**Figure 1:**
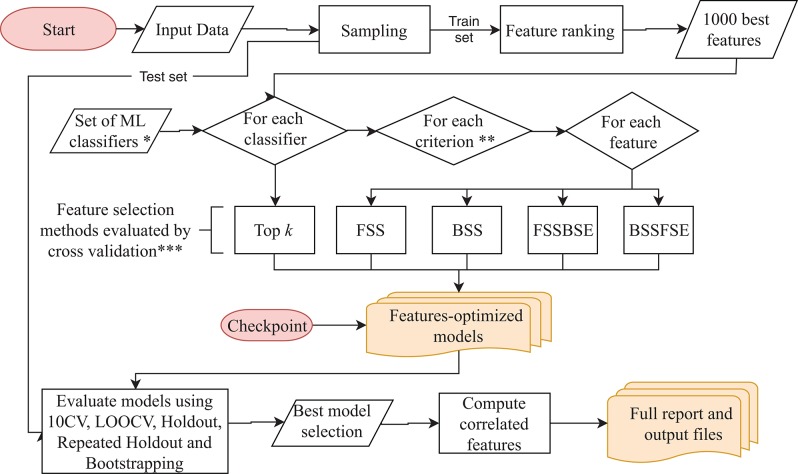
BioDiscML pipeline. Preprocessing and feature selection procedures are fully parallelizable, When all features-optimized models are computed, the model selection starts. The program can be also started from the checkpoint at any moment during the execution. *The Set of ML classifiers is the set of pre-configured commands in classifiers.conf file. All classifiers are listed in the Supplementary Table S1. **Criterions are optimized metrics, evaluated by 10-folds cross validation (10 CV), used to assess if a model is improved, such as accuracy, balanced error rate, Matthew's correlation coefficient, area under the curve, sensitivity, specificity, Root Mean Squared Error, etc. (see Evaluation Criterion). ***Feature selection methods include forward stepwise selection (FSS), backward stepwise selection (BSS), forward stepwise selection and backward stepwise elimination (FSSBSE), backward stepwise selection, and Forward stepwise elimination (BSSFSE), and “top **k**” features (see Optimal Feature Subset Search Methods).

BioDiscML pipeline presented in Figure 1 works as follows: It starts with the preprocessing section. After merging the input datasets when many are submitted, a first sampling step separates the data in a train and a test set (2/3 and 1/3, respectively, by default), this latter will be used after model creation to assess non-overfitting. Then, a feature ranking algorithm sorts the features based on their predictive power with respect to the class. Only the first best 1,000 s features are kept by default. Then, in the feature selection section, for each machine learning algorithm defined in BioDiscML (i.e., the classifiers), and for each optimization evaluation criterion (i.e., a chosen evaluation metric), two types of feature search selection are performed: top *k* features and stepwise (see Optimal Feature Subset Search Methods). Top *k* simply select the best *k* elements from the ordered feature set to create a model. In the stepwise approaches, for each element in the ordered set, features are added and/or removed one by one depending on the feature search method. At each iteration, the created model is evaluated by 10-fold cross validation (10 CV) and the combination of selected features is retained if the predictive performance is improved. When all features are tested and the signature is identified, the model is evaluated on other cross-validation/sampling procedures (see Model Evaluation). Once all classifiers are tested, we end with a set of feature-optimized models with their associated performances metrics (see Model Evaluation) and associated features, for each model. In total, about 8,500 models for classification and about 1,800 for regression are tested, but a large part will not be computed because of non-supported data (see Supplementary Table S1). Once all models are generated, the program executes the best model(s) selection section. The average performance among some computed metrics (see Model Evaluation) are used to estimate the most efficient model (see Best Model Selection), and correlated features are retrieved from the original dataset (see Correlated Features Search) and compiled in a tabular-separated text file report. Depending computing performances and dataset size, a few hours may be needed for BioDiscML pipeline to finish. Before the end of BioDiscML execution, a user can execute at any time BioDiscML from the checkpoint in parallel to perform the best model selection process, which will retrieve models from the feature-optimized model list generated and updated in real-time.

#### Data Preprocessing

**Figure 2:**
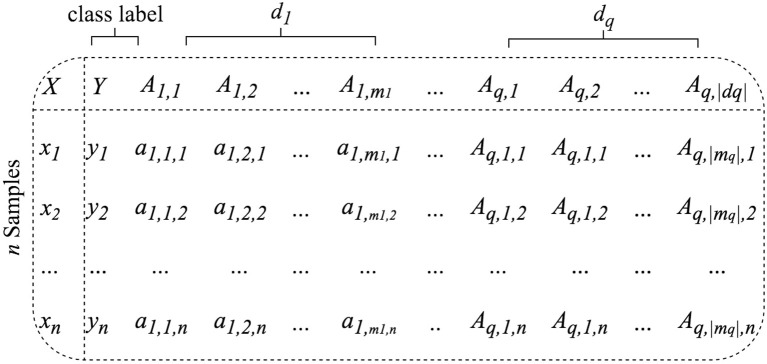
BioDiscML accepts as input one (**{*d***_**1**_**}** only) or many (**{*d***_**1**_**, ..,*d***_***q***_**}**) symbol-separated table-like structured datasets containing samples in row and features in columns.

BioDiscML supports multiple input files (e.g., clinico-pathological information with omics data), as the condition that sample identifiers exist in all files to perform joining. The input datasets are assumed to be clean and consistent, in a flat file format, table-like structure with samples in rows and features in columns (Figure 2). Field separator symbols (e.g., tabulation, comma, semicolon) are automatically detected based on the first lines of the file. Feature and instance duplicate names are not allowed. Where multiple datasets are submitted, only one must contain the class label. File contents are composed of instance identifiers (e.g., samples, patients) associated to numerical and/or nominal features (e.g., high/medium/low, effect_A/effect_B, Drug_1/Drug_2). Let be a set of *q* datasets {*d*_1_, *d*_2_, …, *d*_*q*_}with *q*≥1containing *m*_*q*_features. In each dataset the first column is used to create the joining of all datasets and consists of instances unique identifiers. If an identifier does not exist in all datasets, it will be ignored. The class label column Y is required and must be specified by the user. In addition to the class label, the dataset *d*_1_contains a set of *m*_1_ features noted *A*_1_ = {*A*_1, 1_, *A*_1, 2_, …, *A*_1,_*m*__1__}where *A*_1,_*m*__1__, the *m*_1_-th feature of *d*_1_, is a vector denoted {*a*_1,_*m*__1_, 1_, *a*_1,_*m*__1_, 2_, …, *a*_1,_*m*__1_, *n*_}. Hence the feature vector of the *n*-th instance of the dataset *d*_1_ is noted *x*_1, *n*_ = {*y*_*n*_, *a*_1, 1, *n*_, *a*_1, 2, *n*_, …, *a*_1,_*m*__1_, *n*_}.In case of multiple datasets (*q*≥2), the feature vector of the *n*-th instance of the dataset *d*_r_ is noted then *x*_*r, n*_ = {*a*_*r*, 1, *n*_, *a*_*r*, 2, *n*_, …, *a*_*r*,_*m*__*r*_, *n*_}, where *m*_*r*_ is its number of features. The resulting set of merged datasets is called *D*.

Due to experimental errors or partially answered forms by patients, missing data may be present in the dataset. If one wants to conserve the features with missing data, the ML library used by BioDiscML will replace all missing values for nominal and numeric features with the modes (i.e., value that occurs most often) and means from the training data, respectively.

Also, manipulating large files is painful and one would exclude specific features without editing the input files. Thus, we implemented in BioDiscML features exclusion capabilities, where it simply ignores columns entered by the user.

Finally, a stratified sampling, which preserve the initial classes balancing, is applied to generate a test set for further evaluation to assess non-overfitting. It is set by default to create a train set of 2/3 of the input data, from which models will be computed, and 1/3 as a test set. These proportions can be modified by the user, and in case of very low number of instances, sampling can be disabled. A separate test set of the same structure than the train set can also be provided to BioDiscML.

#### Feature Ranking and Dimension Reduction

Feature ranking (as for feature selection) is essential to identify irrelevant or redundant features, which, once discarded, help to reduce computation time, improve prediction performance, and extract the most informative features (Sasikala et al., 2016). BioDiscML uses Information Gain (Krishnaiah and Kanal, 1982), which evaluates the worth of a feature by measuring the information gain with respect to a class. However, Information Gain is not compatible for regression problems using continuous class. In this case, BioDiscML instead uses ReliefF (Robnik-Sikonja and Kononenko, 1997), an adaptation to the original Relief algorithm (Kira and Rendell, 1992), which is as fast as Information Gain computation. ReliefF evaluates the worth of a feature by repeatedly sampling an instance and considering the value of the given feature for the nearest instance of the same and different class. Both Information Gain and ReliefF are used in conjunction with a ranker search algorithm, which ranks features by their individual evaluations. By default, and to reduce the dimension of the dataset, BioDiscML will only keep informative features (Information Gain >0.01 or |ReliefF| >0.01) or the first 1,000 best features, ordered by their absolute value of their score (ReliefF provides positive and negative correlation scoring with continuous class) (see Algorithm 1).

**Algorithm 1:** Dimension reduction by Information Gain and ReliefF

**Input**: train instances of *D* (merged datasets), *classifierType*(classification or regression)
**Output**: Dataset with ranked best features *S*

**for each** feature array *A* **do**
**if** *classifierType* = classification
**then** *meritScore*_*a*_ = Compute information gain
value of *A* with respect to classes *Y*
**else** *meritScore*_*a*_ = Compute ReliefF value of *a*
with respect to classes *Y*
**end if**
**if** *meritScore*_*a*_≠0
**then** add | *meritScore*_*a*_ |to *meritScores*
**end if**
**end for**
*SortedFeatures* = Sort *meritScores* from largest to smallest values
**if** |*SortedFeatures*| ≤ 1000
**then** *S* = *SortedFeatures*
**else** *S* = *SortedFeatures*{*A*_1_, *A*_2_, …, *A*_1000_}
**end if**
**return** *S*

#### Feature Subset Selection and Model Search

Selecting a subset of features from a large number of potential variables is a common problem in pattern classification. Some feature subset selection methods involve a criterion to evaluate the capacity of feature subsets to distinguish one class from another, and a search algorithm to explore the potential solution space. At the end of the process, the feature subset generally contains the most important and non-redundant variables. In this context, BioDiscML automates an exhaustive procedure that generates thousands of combinations of ML algorithms and feature subsets defined by various search methods. This technique, which mixes both feature and model search, produces thousands of models associated to an optimal subset of non-redundant features. Many evaluation procedures (e.g., cross validations, resampling, bootstrapping) using train and test sets assess if models do not overfit the train set. All steps are described in Algorithm 2.

**Algorithm 2:** Identification of features subsets and feature-optimized models

**Input**: Dataset with ranked best features *S*, set of ML *classifiers with various hyperparameters, set of *criteria*, datasets *D**
**Output**: Feature-optimized models list *L* with their identified features subset

**function** EVALUATE(*model, selectedFeatures*, dataset *D*, list of models *L*)
*trainSetEvaluation* = Evaluate *model* using 10CV, LOOCV, Bootstrap, Repeated Holdout, 0.632+ estimator on train set
*testSetEvaluation* = Extract *selectedFeatures* from test instances of dataset *D* and perform holdout evaluation with *model*
*performances* = *trainSetEvaluation, testSetEvaluation* add *model* with *performances* and *selectedFeatures* to *L*
**return** *L*
**end function**
**for each** *classifier* in *classifiers* **do**
**for each** *criterion* in *criteria* **do**
**for each** *featureSearchMethod* in f*eatureSearchMethods{FSS*, BSS, FSSBSE, BSSFSE)
**do**
**if** *criterion* must be maximized
(see Supplementary Table S2) **then**
*criterionScore* = *0*
*rule* = “lesser than”
**else**
*criterionScore* = *1000*
*rule* = “greater than”
**end if**
**if** *featureSearchMethod* = *FSS* or *BSS* **then**
**if** *featureSearchMethod* = *BSS* **then**
*S* = invert feature rank order of *S*
**end if**
**for each** feature *A* in *S* **do**
Add *A* to *selectedFeatures*
*model* = Train using *classifier* with *selectedFeatures*
*newCriterionScore* = perform 10CV evaluation
**if** *newCriterionScore rule CriterionScore*
**then** discard *a* from *selectedFeatures*
**else** keep a in *selectedFeatures*
*criterionScore* = *newCriterionScore*
**end if**
**end for**
**else**
**if** *featureSearchMethod* = BSSFSE
**then** *S* = invert feature rank order of *S*
**end if**
**for each** feature *A* in *S* **do**
Add *A* to *selectedFeatures*
*model* = Train using *classifier* with *selectedFeatures*
*newCriterionScore* = perform 10CV evaluation
**if** *newCriterionScore rule CriterionScore*
**then** discard *A* from *selectedFeatures*
**else**
keep *A* in *selectedFeatures*
*criterionScore* = *newCriterionScore*
**for each** *selectedFeature* from before last kept
feature to the first selected feature in
*selectedFeatures* **do**
remove *selectedFeature* from *selectedFeatures*
*subModel* = Train using *classifier* with *selectedFeatures*
*subNewCriterionScore* = perform 10CV evaluation
**if** *subNewCriterionScore rule*
*NewCriterionScore* **then**
discard *selectedFeature* from *selectedFeatures*
*NewCriterionScore* = *subNewCriterionScore*
**else**
keep *selectedFeature* in *selectedFeatures*
**end if**
**end for**
**end if**
**end for**
**end if**
*L* = EVALUATE(*model, selectedFeatures, A, L)*
**end for**
**end for**
# create models without stepwise feature subset selection approaches
*selectedFeatures* = *k first* features
*model* = Train using *classifier* with *selectedFeatures* from dataset *S*
*L* = EVALUATE(*model, selectedFeatures, A, L)*
**end for**
**return** *L*

##### Available machine learning algorithms

ML classifier algorithms and their hyperparameters (i.e., the options of the learning algorithm) are predefined in BioDiscML with random sets of options, including those provided by default in Weka library. In the current version, about 80 classifiers are available in BioDiscML (Supplementary Table S1). Some classifiers exist in various adaptations to support more features or class types. Depending available computing resources, the list of classifiers and hyperparameters can be modified by the user, as well as the spectrum of tested algorithms. In case of non-compatibility between a classifier and the input data or erroneous options, the classifier will be ignored by BioDiscML.

##### Evaluation criterion

For each classifier, several feature search methods are conducted. Each search method iterates over the features (except “top *k*” features approach) and trains a model at each iteration. To evaluate if a model is improved by adding or removing a feature, an evaluation criterion is measured by 10-fold cross-validation to assess if the prediction performance increases. All metrics are averaged over the folds and by class size, since a classifier usually performs differently over each class. This optimization procedure performed on feature selection either maximize or minimize the criterion, depending if it measures a performance or an error, respectively. Criterions supported by BioDiscML includes accuracy (ACC), balanced error rate (BER), Matthew's correlation coefficient (MCC), area under the curve (AUC), sensitivity, specificity, Root Mean Squared Error (RMSE), Correlation Coefficient (CC), etc. The full criterions list, including their equations, is provided in Supplementary Table S2.

##### Optimal feature subset search methods

For each ML algorithm listed in Supplementary Table S1, and for each selected criteria selected in Supplementary Table S2, from the ranked features *S* obtained in Algorithm 1, models are trained using several feature search approaches, including: Forward stepwise selection (FSS), Backward stepwise selection (BSS), Forward stepwise selection and Backward stepwise elimination (FSSBSE), Backward stepwise selection and Forward stepwise elimination (BSSFSE), and “top *k*” features. In the stepwise procedures, features having an equal predictive power to the outcome (i.e., distributions similar among classes) and retained in the model may be selected randomly or by order of appearance in the dataset.

*Forward stepwise selection (FSS)*. Also called sequential forward selection (Reunanen, 2003), where features are added one by one to the model. At each added feature, the model is evaluated by 10 CV. If the model is improved, based on a given evaluation criterion, the feature is definitely kept in the model, otherwise it is rejected (Maugis et al., 2011).

*Backward stepwise selection (BSS)*. This approach is similar to the FSS, but instead of starting from the best feature, this algorithm starts the selection from the worst feature. Features are added one by one, if the model is improved (evaluated by 10 CV) the feature is definitely kept in the model, else, it is rejected.

*Forward stepwise selection and backward stepwise elimination (FSSBSE)*. The drawback of FSS and BSS is that once a feature is selected, it cannot be deleted at a later stage. Consequently, redundant features might be selected. To alleviate this problem, we have implemented a FFSBSE algorithm, inspired by previous work (Caruana and Freitag, 1994; Mao, 2004; Zhang, 2011). After each addition of an increasing criterion score feature using FSS, a BSE step removes all previously selected features one by one in reverse order with replacement and test the performance by 10 CV every time. If removing a feature improves the model (evaluated by 10 CV), then the feature is discarded, otherwise it is kept.

*Backward stepwise selection and forward stepwise elimination (BSSFSE)*. Similar to FSSBSE, but instead the algorithm starts from the selection of the worst feature.

*“Top k” features* This fast method simply trains a model with a subset of *k* best features, with *k* = {1, 5, 10, 15, 20, 25, 30, 40, 50, 75, 100}.

##### Model evaluation

Prediction performance of a model is measured using various evaluation procedures including 10 CV, leave-one-out cross validation (LOOCV), holdout, repeated Holdout, bootstrapping, and 0.632+ bootstrap estimator. For each generated model described in previous sections, and for each evaluation procedure, the following metrics are measured (see Supplementary Table S2): ACC, AUC, AUPRC, Sensitivity, Specificity, MCC, BER. In *10 CV evaluation*, the original training set is randomly partitioned into 10 equal sized subsamples. The model is trained on nine subsamples and tested on the remaining one. The CV is repeated 10 times, where each subsample is used exactly once for evaluation. The reported metric scores are their average over all folds. In *LOOCV* each model is trained on all the data except for one instance and a prediction is made for that instance. Average of metric scores are computed over all tested instances. The *holdout* method is the simplest kind of cross validation where the dataset is randomly separated into two sets generated at sampling procedure (see Figure 1), called the training set and the testing set. The model is trained using the training set only, then is used to predict the class for the data in the testing set as evaluation. However, this type of evaluation can have a high variance since it depends heavily on which instances end up in the training and test sets. Thus, a *repeated holdout* is also performed 100 times (by default) with random sampling without replacement. *Repeated Holdout* consists of randomly select and hold out a 1/3 of the training sample for testing, build model with only the remaining samples, retrieve its performances, and repeat the process many times. At the end, we report the average all performance metrics. The *bootstrapping* is equivalent, except the random sampling is performed with replacement. Finally, we also provide a 0.632+ bootstrap estimator (Efron, 1983), representing an estimation of the bias of the predictive model, which should tend to 0, hence assessing that the model does not overfit.

In addition to all these metrics, for each feature-optimized generated models, we calculate the average MCC and BER with their associated standard deviation across all evaluations (10 CV, LOOCV, Repeated Holdout, Bootstrap, holdout). For regression, we calculate the average and standard deviation of CC and RMSE.

#### Best Model Selection

Selecting the best model is not trivial since several good solutions are produced. Moreover, the definition of a “good” model also depends of user needs; for example, one would favor a model with a very low number of features over a model having dozens of feature, even if the latter provides a better overall performance. While BioDiscML proposes an automatic selection of the best model, a manual approach would be appropriate at that step. For this reason, all models are stored in real time in a Microsoft Excel-compatible Comma Separated Value (CSV) file and can be easily ordered by a criterion metric according to the user needs. Identifiers of user-selected models can be then submitted to BioDiscML to generate data files for easy re-use in other programs and full reports (containing the biomarker signature, the model and its hyperparameters, overall performances, and correlated features). Otherwise, by default, BioDiscML best model selection procedure aims to identify the model having a high agreement between the various evaluation methods, hence assessing stability and low overfitting of the model. To this purpose, select the model having the best average MCC with a standard deviation lower than 0.1 (or another adjusted threshold set by the user). The user can change the best model selection strategy at ease in the program configuration file. For example, one would select a trained model on train set having the best MCC on the test set (TEST_MCC, see readme program file), or on the best bootstrapping using merged training and testing sets (TRAIN_TEST_BS_MCC).Since all generated models have a unique identifier, one would use these identifiers to select the best model based its own criteria.

#### Ensemble Learning

Since several good models with different features can exist in the results generated by BioDiscML, we also propose a vote classifier able to combine many models together. Different combinations of probability estimates for classification are available, including Average of probabilities, Product of probabilities, Majority voting and Median. As for best model selection, many metrics and correlated features are provided for this ensemble model. We also count the number of occurrences of each features in the combined models. The models to add in the ensemble classifier are dependent of the user choice. They can be selected manually using their unique identifiers, or by setting a metric dependent rule (by default average MCC lower than 0.6) and a maximum number of models to include.

#### Correlated Features Search

The identified signatures by stepwise search methods will tend to ignore all redundant/correlated features. To use the models as “black box” for pure prediction, this may be optimal, but not for biological interpretation because one would understand why the selected features have a link with the predicted class. To this purpose, from the features in the signature, BioDiscML retrieves all other correlated features from the original dataset using Pearson and Spearman correlations. BioDiscML also identifies all neighbor features discovered during feature ranking procedure by Information Gain and ReliefF methods. Both provide feature ranking scores that are used to detect the features having the same predictive power, i.e., similar behavior among instances. With these techniques, redundant information lost during the feature selection process are recovered, hence helping for further interpretation of the signature.

#### Gene Set Enrichment Analysis

We performed several Gene Set Enrichment Analysis (GSEA) to characterize the signatures identified by BioDiscML on the test datasets. To this purpose, we used ToppFun tool, from ToppGene suite (Chen et al., 2009), with Bonferroni correction at 0.05 to the probability density function (*p*-value Method).

### Datasets for Benchmarking

Datasets described in Table 1 have been evaluated to compare the performance of BioDiscML and recent tools. All models and signature information for all tested datasets are presented in Supplementary Datasets_results.xlsx.

**Table 1:** Description of the real-world datasets used to evaluate the performance of BioDiscML vs. recent tools.

**Name**	**Description**	**Features**	**Instances**	**References**
Stem cells	Fifteen merged transcriptomics microarray sets from multiple platforms. They contain three types of human cells as classes: human Fibroblasts (Fib), embryonic stem cells (ESC), and induced pluripotent stem cells (IPSC)	13,315	Train set: 62 ESC, 105 IPSC, 43 Fib Test set: 33 ESC, 77 IPSC, 22 Fib Total: 210 (train) + 132 (test) = 342 patients	Rohart et al., 2017a
Colon cancer	Transcriptomics microarray available from ColonCA R package in Bioconductor (Gentleman et al., 2006), separated between cancerous from non-cancerous colon tissue	2,000	Sixty-two patients, including 40 tumors and 22 normal cases	Alon et al., 1999
Central nervous system	Microarray gene expression data derived from central nervous system of patients brain tumors to predict embryonal tumor outcome	7,129	Sixty patients, including 39 medulloblastoma survivors, and 21 treatment failures cases	Pomeroy et al., 2002
Diffuse large B-cell lymphoma (DLBCL)	Transcriptomic microarray of pre-treatment biopsies tumor specimens separated in DLBCL and follicular lymphoma	2,647	Seventy-seven patients, including 58 DLBCL and 19 follicular lymphoma	Shipp et al., 2002
Prostate cancer	Microarray expression analysis was used to determine gene expression levels differences between tumor and non-tumor prostate samples	2,135	One hundred two patients, including 52 tumor and 50 normal cases	Singh et al., 2002

## Results

We compared BioDiscML to various recent approaches dedicated to biomarker discovery and modeling, including MINT (Rohart et al., 2017a), AucPR (Yu and Park, 2014), and RGIFE (Swan et al., 2015) to demonstrate the better predictive performances that BioDiscML offers on various omics datasets. In all cases, BioDiscML outperform these state-of-the-art tools.

### BioDiscML vs. Mint

**Figure 3:**
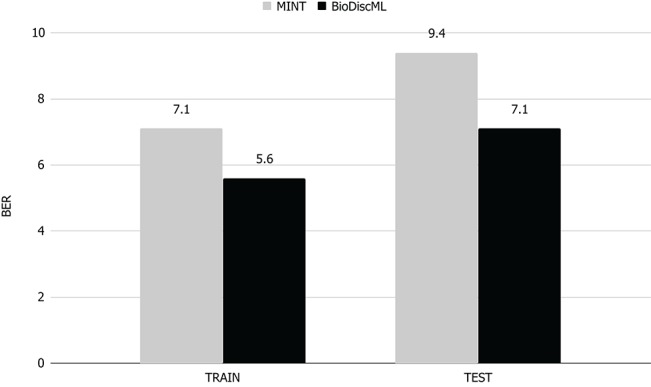
BER comparison of MINT vs. BioDiscML. Train BER value was obtained by LOGOCV performance evaluation and test BER value using holdout validation. Values are in percentage.

MINT implements a multivariate integrative method able to integrate independent datasets, reduce batch effect, classify instances and identify key discriminant variables. In their study, they performed a feature selection and classification evaluation of a stem cell dataset. According to their published results, they identified a signature of 17 genes which predicted the test and train sets with a BER of 9.4 and 7.1% resp. Using the exact same train set, BioDiscML identified a signature of 19 genes by optimizing the AUC of a Random Forest model with 100 iterations and using the FSSBSE feature search method. The measured BER on the test set was 7%, and on the train set 3.5, 3.6, 6.8, and 7.2% using 10 CV, LOOCV, and repeated holdout and bootstraping resp. To select this model among the 4,710 successfully generated models, we simply retrieved the one having the lowest BER on the holdout method. Thus, on the same test set, the Random Forest model identified by BioDiscML improved the BER from 9.4 to 7%, corresponding to about 25% relative error decrease (see Figure 3).

In their paper, MINT authors have provided the signature identified by their method. Although both signatures found by MINT and BioDiscML have no genes in common, most of level 2 biological processes ontologies (see Supplementary Data) obtained by these signatures were identical (cellular process, multicellular organismal process, metabolic process, biological regulation, cellular component organization or biogenesis, localization). Specific biological processes were reproduction and immune system in MINT signature, and response to stimulus and developmental process in BioDiscML signature. A long signature of 71 genes can also be obtained using correlated feature search in BioDiscML. Using this long signature, only immune system process was added compared to the short signature, which also exists in the MINT signature. Moreover, this long signature provided perfect predictions on all instances of the test set. We also compared both signatures GSEA (see Methods). MINT signature did not show any significantly enriched ontologies, literature co-citation, co-expression etc. At the opposite, the short signature of BioDiscML found about 20 hits related to stem cells in co-expression databases (GeneSigDB and MSigDB) and co-expression Atlas. Also, about 20 other hits were found in literature co-citation about cognitive diseases (Alzheimer, Parkinson, Schizophrenia). The long signature provided even more hits, in many other categories.

### BioDiscML vs. AucPR

**Figure 4:**
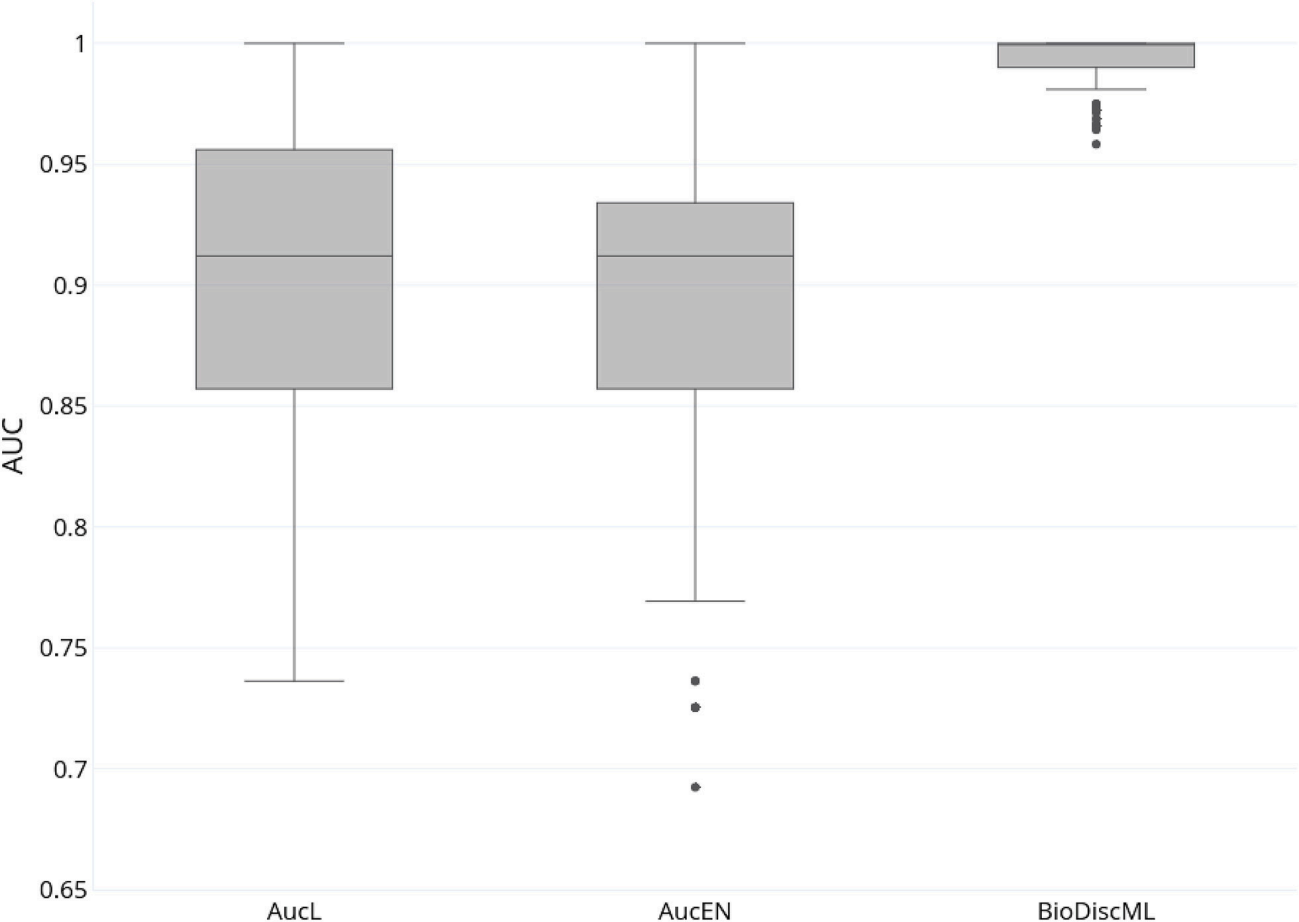
Boxplot of AUCs bootstrapping over 100 iterations of most performant AucPR methods called AucL (AucPR with Lasso) and AucEN (AucPR with ElasticNet), vs. BioDiscML most performant model (Hoeffding Tree).

In their study, authors of AucPR, an AUC-based approach using penalized regression, have evaluated the performance of their tool against four datasets. While AucPR showed a very good prediction performance on three of four tested datasets, the average AUC on *ColonCA* dataset was about 90% using both best penalization regression approach modes of the tool (Lasso and ElasticNet). Considering AucPR had the lowest performance on this dataset, we tried the performance of BioDiscML on it. In their paper, authors report the boxplots of 100 AUCs obtained by repeated holdout (random separation of 2/3 of the data for training and the remaining for testing) without sampling step. Using the same data and same evaluation method without sampling before training, two models identified by BioDiscML, on the 3,967 successfully generated models, shared the same best average AUC score. We chose the one having the best MCC on repeated holdout, a model based on a Hoeffding Tree (parameters: infogain split, Naive Bayes adaptive leaf prediction strategy, grace period of 200, tie threshold of 0.05) optimized by AUC. This model provided an average AUC of 99.3% (0.632+ rule at 0.047) using 10 genes discovered by FSSBSE. This is an improvement of AUC of about 11%. Both AucPR modes AucL and AucEN selected in comparison 30 and 22 genes resp. The benchmark comparison of AUCs is reported in Figure 4. The model identified by BioDiscML has a much better performance in terms of average AUC and variance over bootstrapping. GSEA was not performed since this dataset didn't provided gene identifiers.

### BioDiscML vs. RGIFE

RGIFE is an heuristic method intending to identify reduced panels of biomarkers with highly predictive performance. It first ranks features by their contribution to the generated models, and dynamically removes blocks of features. It also introduces a concept called soft-fail, which considers an iteration successful despite a performance drop within a tolerance level and specific circumstances. We evaluated the performance of BioDiscML on three datasets tested in RGIFE, including Central Nervous System (CNS), DLBCL, and Prostate Cancer datasets. On the 10 tested datasets by RGIFE, the three selected datasets showed accuracies around 60–70% for 10 CV, while BioDiscML identified models and signatures providing prediction performance close to perfection (100% accuracy) with lower number of features. Performances are reported in Table 2, where, for each dataset, we identified two models found by BioDiscML. To provide a fair comparison with the RGIFE manuscript we selected models having the best 10 CV accuracy (with best bootstrapping accuracy and lowest number of features in case of models' performance equality), which ended with 100% accurate models. But since this typical measure approach tends to be over-optimistic on the real performance of the models and because overfitting was suspected, we also reported models having the best bootstrapping accuracy. Obtained models show accuracies between 10CV and Bootstrapping more consistent, hence showing models are stable. In any case, 10CV accuracy was always better with BioDiscML results. The two signatures found for CNS dataset presented an overlap of five genes, and a merged list of the signatures show several GSEA significant hits related medulloblastoma and other cancers. For BLBCL dataset, no genes overlapped the two signatures, and we found significant hits related to dehydrogenase activity in the GSEA analysis on the merged list of the signatures, which has a link with follicular lymphoma to diffuse large B-cell lymphoma (Montoto et al., 2007). Finally, the prostate cancer signatures showed no overlap either, but GSEA analysis on the merge lists show several hits related to this cancer.

**Table 2:** Performances of RGIFE vs. BioDiscML measured by accuracy obtained through 10-fold cross validation (10CV_ACC) and bootstrapping (BS_ACC).

	**RGIFE**			**BioDiscML**					
**Dataset**	**10CV_ACC**	**#Features**	**Model**	**10CV_ACC**	**BS_ACC**	**#Features**	**Model**	**Search**	**Criterion**
CNS	77.1	Not reported	KNN	100	80.7	12	A2DE	BSSFSE	AUC
				93.3	98.6	11	HT	FSSBSE	AUC
DLBCL	68	9	RF	100	93	6	A1DE	FSSBSE	MCC
				98.7	98.3	6	NB	FSSBSE	AUC
Prostate cancer	95.2	158	SVM	100	91	12	VFI	BSSFSE	ACC
				99	95.7	10	NB	FSSBSE	AUC

*Classifiers evaluated by RGIFE were K-Nearest Neighbors (KNN), Random Forest (RF), and Support Vector Machines (SVM). Most performant classifiers identified by BioDiscML were Average two Dependance Estimators (A2DE), Hoeffding Tree (HT), Average 1 Dependance Estimators (A2DE), Voting Features Intervals (VFI), and Naive Bayes (NB). Hyperparameters are described in Supplementary Data. Various criteria were used, including AUC, MCC, and FDR, and two feature search BSSFSE and FSSBSE. The signatures are shown in Supplementary Data*.

In terms of computing performances, on a same server containing four Intel(R) Xeon(R) CPU E5-2695 v2 @ 2.40GHz (48 threads), BioDiscML runtime was 28, 387, and 393 min on CNS, DLBCL, and Prostate Cancer datasets resp., and generated 5,751, 6,479, and 6,408 models resp., without exceeding 16 GB memory usage. In comparison, computation time reported by RGIFE in their Supplementary Data show ranges about 180–400 min.

## Discussion

### A Simplified but Customizable Automated ML Tool

BioDiscML tool has been developed to enhance biomarker discovery using an exhaustive ML approach and propose automation of ML steps to perform such task. A large variety of algorithms is available and combinations of strategies are countless if we consider the hyperparameters of all classifiers and feature selection algorithms. This complexity is a barrier to non-expert users attempting to use ML to analyze their data. Thus, we designed BioDiscML to simplify ML steps without penalizing the performance, such as using fast and optimal feature ranking algorithms and feature search methods, limit the number of features after feature ranking, and establish predefined classifiers hyperparameters to reduce computing time. Although editable in BioDiscML configuration file, these intentional limitations provide researchers a program that generate results without intervention within a few hours of calculation on a recent computer.

### A Sampling Procedure to Avoid Overtfitted Models

BioDiscML implements a sampling step to assess the non-overfitting and the good performance of identified models and signatures, where it splits the dataset into two stratified (class balancing is preserved) random parts. The program also accepts a second input file as a test dataset, as long as it is in the same format as the train set. In case of very limited instances, it is possible to skip the sampling operation, although not recommended because of the risk to not detect overfitted models. A reasonable number of instances (i.e., samples) should be provided to BioDiscML, else it is expected to obtain models with low performances. For example, we estimate that a highly heterogeneous dataset, such as prostate or breast cancer data, should contain at least half-hundred patients per class, while a dataset based on a study involving cloned living species could be limited to half a dozen individuals per class.

### Feature Selection Procedures in BioDiscML Are Fast and Scalable

Omics datasets are generally composed of a thousands of features. To simplify input datasets and save computation time BioDiscML implements a feature ranking and dimension reduction procedure. Many approaches exist (Chandrashekar and Sahin, 2014) and most are applicable to biological problems (Saeys et al., 2007), but we choose to only implement Information Gain (Krishnaiah and Kanal, 1982) for classification, and ReliefF (Robnik-Sikonja and Kononenko, 1997), for regression, since they are fast and highly scalable univariate tests (Saeys et al., 2007). Information Gain shown very good performance on biological data (Li et al., 2004, 2011; Abusamra, 2013), as for ReliefF (Marchiori et al., 2005; He and Yu, 2010; Wang et al., 2016). Besides, their ranking capability provides an easy way to eliminate redundant, non-informative and noisy information, hence our choice to provide only those in BioDiscML.

### BioDiscML Uses All Available Classifiers From a Widely Accepted and Efficient ML Library

There is a plethora of ML algorithms specialized in classification (i.e., categorical class) and regression (i.e., continuous class). BioDiscML covers many of them but can also be manually limited to the most known and widely applied in biomedical research for the development of predictive models such as Random Forest, Decision Trees, Rules, Naive Bayes, Artificial Neural Networks, Bayesian Networks and Support Vector Machines. They all resulted in effective and accurate decision-making (Jagga and Gupta, 2015). But the final models created with these classifiers in various studies were all delivered after an exhaustive search work. BioDiscML aims to reduce this search time by providing the models adapted to user datasets. All ML algorithms are provided by an advanced freely available ML library toolkit, called Weka. Besides this library, various ML libraries exist, such as SciKit-Learn (Nelli, 2015) (written in Python) and packages in R (Lesmeister, 2017). BioDiscML implements Weka library for various reasons, including its wide usage in computational biology (Gewehr et al., 2007; Bendl et al., 2014; Bernardi et al., 2015; Arganda-Carreras et al., 2017; Chicco, 2017; Alves et al., 2018), its high citation rate (at August 2018) and its highly versatile object-oriented language JAVA (e.g., easy to parallelize, multi-platform compatibility, GUI integration, generally already installed on clients, etc.), which is much faster (Fourment and Gillings, 2008) and scalable than Python or R. Finally, the user can use Weka GUI (graphical interface) to explore BioDiscML results, generate ROC curves or try other combinations of classifiers by hand. For example, the output files generated by BioDiscML are compatible with Weka and can be loaded in its GUI.

### A Combination of Model Search and Feature Search Procedures to Identify Highly Predictive Models

BioDiscML combines the model search and the feature search together to identify biomarker signatures. Using the various search methods (i.e., stepwise and top *k*) and optimized criteria, each model is associated to a signature of features. Forward and backward stepwise search methods return signatures that are optimized on the classifier and the criterion. Note that the backward stepwise search approaches (BSS, BSSFSE) are not the usual “backward elimination” used in the literature (Sutter and Kalivas, 1993) for variables selection since it would be computationally expensive here. Instead, backward selection starts from worst features and will generally return performant models only when most of features have a relatively good univariate information gain or ReliefF score. The signature then reveals a combination of biomarkers which, associated together in a model, provide a highly predictive value of the class.

To assess the overall performance of the models, their robustness and the absence of overfitting, various well-known evaluation methods (Arlot and Celisse, 2010) have been implemented in BioDiscML, because some may not be adapted to all situations. For example, for biomedical studies which generally produce a low number of patients (i.e., instances), bootstrapping is a good alternative to sampling (Chen et al., 2002) (i.e., split in train and test set, involving waste of data). Besides, it is known that *k*-fold cross validation tends to deliver over-optimist performances (Smith et al., 2014). To facilitate the choice of the best models, we provide many performance metrics that can be averaged over all evaluation methods. BioDiscML also provide an ensemble classifier based on a voting system to include many models with different signatures. This method is known to provide better predictive performance than could be obtained from any of the constituent learning algorithms alone (Polikar, 2006).

### Signature Interpretation Is Still a Challenge

A biologist will want to interpret and validate *in silico* the signature, since there is an obvious relation between the identified biomarkers in a signature and the predicted class (e.g., outcome). To perform such task, there exist many Gene Set Enrichment Analysis (GSEA) tools, such as ToppGene suite (Chen et al., 2009) or Enrichr (Kuleshov et al., 2016). These GSEA tools will provide a characterization of signature and confirm to the biologist if the signature has a biological meaning with the original study from which the dataset have been generated. Some more extensive literature searches may provide more insights and help linking the signatures' features with the predicted class.

Moreover, in some cases, the biologist, based on its experience and knowledge, may not find the biomarkers he expects in the signatures. This is a consequence of the feature search procedure which produces highly optimized signatures. This optimization tends to ignore all redundant features that could potentially help the biological interpretation of the biomarkers related to the class. To overcome this issue, BioDiscML retrieves all correlated features that could have been excluded during the feature subset selection and model search procedure. It is important to note that adding signature's perfectly correlated features (100% correlated) to the model will maintain its performance. At the opposite, it is expected to have a slight performance drop when adding “almost-correlated” features (95–99% correlation), which can be tested by training and evaluation of the model with the added correlated features.

Some scientific visualization tools would have probably been welcome in BioDiscML, but JAVA visualization libraries are rare. However, to overcome this lack, BioDiscML generates a subset of the input dataset containing only the sample values of the signature' features. This subset in comma-separated values format can be loaded easily in other visualization software such as Microsoft Excel, Orange (Demšar et al., 2013), RapidMiner (Hofmann, 2016), or R (Gardener, 2012) to generate heatmaps or boxplots.

### BioDiscML Exhaustive Approach Outperforms Recently Published Tools

We benchmarked BioDiscML against recent tools proposing different approaches to discover biomarker signatures. Benchmarks showed that BioDiscML outperforms these state-of-art methods using same datasets. Because of its exhaustive approach, it was able to identify one or more models with smaller signatures providing much better prediction performances. We also demonstrated in the case of the stem cell dataset that BioDiscML signature contained different genes but similar ontologies than the MINT signature, with a better prediction performance. A GSEA also showed that the BioDiscML signature had much more biological evidence, denoted by the occurrence of stem cells topics in the co-expression databases. The genes in the BioDiscML signatures were also present in neurodegenerative diseases, highlighting the link of these genes with the neuronal system, supported by evidence of efficient stem cell-based therapies for neural repair (Volkman and Offen, 2017). For the other benchmarked datasets which contained gene references, the GSEA analyses also showed supporting evidences assessing the biological relation between the genes found in the signatures and the biological experiment from where they were produced.

It is important to note that short but still very predictive model' signatures can be extended as an “enriched” signature which include the correlated genes. These enriched signatures may increase the accuracy of the signature, but more importantly they can help to better understand the biological meaning of the model. On the MINT dataset, BioDiscML showed a perfect prediction on the test set with the enriched signature and retrieved more ontologies.

Finally, in this paper we benchmarked BioDiscML only on transcriptomics datasets from microarray data provided by the tools we tested. But BioDiscML showed also good performances in other omics datasets tested in other contexts (data not shown).

### Performant Models Identified in Minutes

BioDiscML computing performances are highly dependent on the size of the input dataset and the available processors. To generate all models implemented in the software, it requires a few hours of computation. However, it is possible to restrict BioDiscML to a specific list of algorithms, hence reducing the computation time to seconds or minutes. It is also possible to extract the best signatures and models produced since the beginning of BioDiscML execution at any time. We have prioritized the training of the most common and fastest classifiers to propose a large number of computed models shortly after starting BioDiscML. More complex models, such as Multilayer perceptrons, are set in low priority. More running time will simply increase the probability to obtain a better model. The user is informed in the command line output the progression of the program (i.e., the number of models trained and remaining to train). Finally, BioDiscML can be stopped at any moment, especially if the user is not interested to let BioDiscML train complex classifiers.

## Conclusions

This paper introduces BioDiscML, dedicated to identify optimal combination of biomarkers (i.e., features) and machine learning models to predict measured outcomes. It provides a user-friendly and powerful solution to researchers in the medical field looking to identify predictive features, essential to the development of personalized medicine approaches and research of new therapeutic targets. This software has the benefit to exploit a large number of machine learning classifiers within a fully automated process combined with data pre-processing, hence facilitating the work of a non-machine learning experts audience. Expert users have also the possibility to configure advanced options. BioDiscML is a great opportunity to reduce biomarkers search time, by revealing the most adapted classifiers to a given dataset and even proposes new algorithms poorly explored in the literature that could have a great potential to classify biological data. Otherwise, although this program has been tested with omics data and proven its better performances compared to recent computational biology tools created for the same purpose, it is compatible with any other non-biological data. Finally, the ML library used in BioDiscML is highly maintained, hence enabling convenient additions of newly implemented algorithms in future versions.

## Data and Software Availability

BioDiscML software project and the datasets analyzed during the current study are available at https://github.com/mickaelleclercq/BioDiscML under GPL-3.0 license. This software written in JAVA is compatible with the main operating systems. Windows, Linux and Mac.

## Author Contributions

ML designed and implemented BioDiscML software, conducted literature searches, researched data and selected relevant articles. ML also created figures and tables, and wrote, formatted and finalized the article for submission. BV were in charge to test the software and report all bugs. MM-M and MS helped to optimize BioDiscML pipeline and the implemented algorithms. OP helped to improve the manuscript during the reviewing process. AB, YF, and AD supervised and reviewed the design of the study. All authors contributed to writing and reviewing the manuscript.

### Conflict of Interest Statement

OP was employed by company L'Oréal. The remaining authors declare that the research was conducted in the absence of any commercial or financial relationships that could be construed as a potential conflict of interest.
