# Supplementary material for: Large-Scale Automatic Feature Selection for Biomarker Discovery in High-Dimensional OMICs Data
Source: Front Genet. 2019 May 16;10:452. doi: 10.3389/fgene.2019.00452 (PMC6532608; doi:10.3389/fgene.2019.00452)
Supplement: Supplementary file 1 [file Data_Sheet_1.docx]

***Supplementary materials***

1. **Supplementary Tables**

**Table S1: Algorithms available in BioDiscML. Supported feature types include nominal data (Nom), Numerical data (Num) and Missing Values (MV). Supported class types include nominal data, binary data (i.e. maximum two classes accepted) and numerical data.**

| **Category** | **Classifier** | **Supported feature types** | | | **Supported class types** | | | **Ref.** |
| --- | --- | --- | --- | --- | --- | --- | --- | --- |
|  |  | **Nom** | **Num** | **MV** | **Nom** | **Bin** | **Num** |  |
| Bayes | Averaged N Dependence Estimators (A1DE, A2DE) | x | x | x | x | x |  | [(Webb et al. 2011)](https://paperpile.com/c/aQ1u2M/ytP5X) |
|  | Bayes Network | x | x | x | x | x |  | [(Bouckaert 2004)](https://paperpile.com/c/aQ1u2M/6xwxZ) |
|  | Complement Naive Bayes |  | x | x | x | x |  | [(Rennie et al. 2003)](https://paperpile.com/c/aQ1u2M/CnN8P) |
|  | Hidden Markov Model | x | x |  | x | x |  | [(Bouckaert 2004; Baum and Petrie 1966)](https://paperpile.com/c/aQ1u2M/6xwxZ+NHlf8) |
|  | Hidden Naive Bayes | x |  |  | x | x |  | [(H. Zhang, Jiang, and Su 2005)](https://paperpile.com/c/aQ1u2M/g8p2I) |
|  | Naive Bayes | x | x | x | x | x |  | [(John and Langley 1995)](https://paperpile.com/c/aQ1u2M/HZl0K) |
|  | Multinomial Naive Bayes |  | x |  | x | x |  | [(Andrew McCallum 1998)](https://paperpile.com/c/aQ1u2M/TY5s0) |
|  | Simple Naive Bayes | x | x | x | x | x |  | [(Duda and Hart 1973)](https://paperpile.com/c/aQ1u2M/GyvM1) |
| Functions | ElasticNet |  | x |  |  |  | x | [(Friedman, Hastie, and Tibshirani 2010)](https://paperpile.com/c/aQ1u2M/Jxnmr) |
|  | Fisher's Linear Discriminant (FLDA) |  | x |  |  | x |  | [(Cristianini 2004)](https://paperpile.com/c/aQ1u2M/PHOLF) |
|  | Gaussian Processes | x | x | x |  |  | x | [(MacKay and Mac 2003)](https://paperpile.com/c/aQ1u2M/5lbEC) |
|  | Isotonic Regression |  | x |  |  |  | x | [(Simone, Marino, and Toraldo, n.d.)](https://paperpile.com/c/aQ1u2M/eeOsI) |
|  | Kernel Logistic Regression | x | x | x |  | x |  | [(Ziqiang Wang, Wang, and Sun 2010)](https://paperpile.com/c/aQ1u2M/9JGZY) |
|  | Latent Dirichlet allocation (LDA) |  | x |  | x | x |  | [(Blei, Ng, and Jordan 2003)](https://paperpile.com/c/aQ1u2M/yJ3vL) |
|  | Least Median Squared linear regression | x | x | x |  |  | x | [(Rousseeuw and Leroy 1987)](https://paperpile.com/c/aQ1u2M/F7kLe) |
|  | LibLinear | x | x | x | x | x |  | [(Fan et al. 2008)](https://paperpile.com/c/aQ1u2M/cGPhj) |
|  | Support Vector Machine LibSVM | x | x | x | x | x |  | [(Chang and Lin 2011)](https://paperpile.com/c/aQ1u2M/XxwnM) |
|  | Linear Regression | x | x | x |  |  | x | [(Yan 2009)](https://paperpile.com/c/aQ1u2M/EvMoh) |
|  | Multinomial Logistic Regression | x | x | x | x | x |  | [(Cessie, Le Cessie, and Van Houwelingen 1992)](https://paperpile.com/c/aQ1u2M/2N4es) |
|  | Multilayer Perceptron (multiple implementations) (MLP) | x | x | x | x | x | x | [(Rosenblatt 1961)](https://paperpile.com/c/aQ1u2M/nmIJH) |
|  | Quadratic Discriminant Analysis (QDA) |  | x |  | x | x |  | [(Cover 1965)](https://paperpile.com/c/aQ1u2M/BmX2S) |
|  | Radial Basis Function (RBF), incl. Network and regressor | x | x | x | x | x | x | [(Frank 2014)](https://paperpile.com/c/aQ1u2M/vEYff) |
|  | Stochastic Gradient Descent (SGD) | x | x | x |  | x |  | [(Theodoridis 2015; Shalev-Shwartz and Ben-David 2014)](https://paperpile.com/c/aQ1u2M/qkqqR+kqYjs) |
|  | Simple Linear Regression |  | x | x |  |  | x | [(von Eye and Schuster 1998)](https://paperpile.com/c/aQ1u2M/CUuMW) |
|  | Simple Linear Logistic regression | x | x | x | x | x |  | [(Sumner, Frank, and Hall 2005)](https://paperpile.com/c/aQ1u2M/wFFqo) |
|  | Sequential Minimal Optimization (SMO) | x | x | x | x | x |  | [(Platt 1999; Keerthi et al. 2001; Hastie and Tibshirani 1998)](https://paperpile.com/c/aQ1u2M/qfNy5+BNw2i+iKQAc) |
|  | Sequential Minimal Optimization regression | x | x | x |  |  | x | [(Shevade et al. 2000; Smola and Schölkopf 2004)](https://paperpile.com/c/aQ1u2M/Gw1MN+Az3yc) |
|  | Stochastic Primal Estimated sub-GrAdient SOlver for SVM (Pegasos) | x | x | x |  | x |  | [(Shalev-Shwartz, Singer, and Srebro 2007)](https://paperpile.com/c/aQ1u2M/CjJbU) |
|  | Voted Perceptron | x | x | x |  | x |  | [(Freund and Schapire 1998)](https://paperpile.com/c/aQ1u2M/2hqLQ) |
|  | Winnow | x |  | x |  | x |  | [(Littlestone 1988)](https://paperpile.com/c/aQ1u2M/4HkbH) |
| Lazy | Analogical Modeling | x |  | x | x | x |  | [(Skousen 1989)](https://paperpile.com/c/aQ1u2M/2XB8z) |
|  | K nearest neighbours classifier with various distance measures | x | x | x | x | x |  | [(Bazan and Szczuka 2001; Wojna and Kowalski 2005)](https://paperpile.com/c/aQ1u2M/Kevc5+WLEmK) |
|  | K nearest neighbours with local metric induction | x | x | x | x | x |  | [(Bazan and Szczuka 2001; Wojna and Kowalski 2005)](https://paperpile.com/c/aQ1u2M/Kevc5+WLEmK) |
|  | K-nearest neighbours (IBk) with and without Gaussians | x | x | x | x | x | x | [(Aha, Kibler, and Albert 1991)](https://paperpile.com/c/aQ1u2M/XA77G) |
|  | KStar | x | x | x | x | x | x | [(Cleary and Trigg 1995)](https://paperpile.com/c/aQ1u2M/drSQh) |
|  | Lazy Bayesian Rules (LBR) | x |  | x | x | x |  | [(Zheng and Webb 2000)](https://paperpile.com/c/aQ1u2M/KWcOK) |
|  | Locally Weighted Learning (LWL) | x | x | x | x | x | x | [(Frank, Hall, and Pfahringer 2002; Atkeson, Moore, and Schaal 1997)](https://paperpile.com/c/aQ1u2M/rjTlq+EcPUa) |
|  | Nearest-neighbour (IB1) | x | x | x | x | x |  | [(Aha, Kibler, and Albert 1991)](https://paperpile.com/c/aQ1u2M/XA77G) |
| Misc. | CHIRP | x | x | x | x | x |  | [(Wilkinson, Anand, and Tuan 2011)](https://paperpile.com/c/aQ1u2M/uUwDr) |
|  | Fuzzy Lattice Reasoning (FLR) |  | x | x | x | x |  | [(Kaburlasos, Athanasiadis, and Mitkas 2007)](https://paperpile.com/c/aQ1u2M/2GCz4) |
|  | HyperPipes | x | x | x | x | x |  | [(Witten et al. 2016)](https://paperpile.com/c/aQ1u2M/g4ZoG) |
|  | Ordinal Stochastic Dominance Learner (OSDL) | x |  |  | x | x |  | [(Lievens, De Baets, and Cao-Van 2008; Cao-Van 2003)](https://paperpile.com/c/aQ1u2M/Pnfqp+ZsbQC) |
|  | Voting Feature Intervals (VFI) | x | x | x | x | x |  | [(Demiröz and Altay Güvenir 1997)](https://paperpile.com/c/aQ1u2M/ihnLA) |
| Rules | ConjunctiveRule | x | x | x | x | x | x | [(Witten et al. 2016; King and Holt 1970)](https://paperpile.com/c/aQ1u2M/g4ZoG+FYpTo) |
|  | Decision Table/Naive Bayes hybrid (DTNB) | x | x | x | x | x |  | [(Hall and Frank 2008)](https://paperpile.com/c/aQ1u2M/veSV1) |
|  | Fuzzy Unordered Rule Induction Algorithm (FURIA) | x | x | x | x | x |  | [(Hühn and Hüllermeier 2009)](https://paperpile.com/c/aQ1u2M/4THfq) |
|  | Repeated Incremental Pruning to Produce Error Reduction (JRip) | x | x | x | x | x |  | [(Cohen 1995)](https://paperpile.com/c/aQ1u2M/eVQVV) |
|  | Lazy Associative Classifier (LAC) | x |  | x | x | x |  | [(Veloso, Jr., and Zaki 2006)](https://paperpile.com/c/aQ1u2M/CO0vM) |
|  | M5 Rules | x | x | x |  |  | x | [(Holmes, Hall, and Prank 1999; Wang, Witten, and University of Waikato. Department of Computer Science 1996; J. R. Quinlan 1992)](https://paperpile.com/c/aQ1u2M/daRbB+HiRLZ+T0eTR) |
|  | MODLEM | x |  | x | x | x |  | [(Stefanowski, n.d.)](https://paperpile.com/c/aQ1u2M/GA2aP) |
|  | Multi Objective Evolutionary Fuzzy | x | x |  | x | x |  | [(Jiménez, Sánchez, and Juárez 2014)](https://paperpile.com/c/aQ1u2M/HA6tj) |
|  | Nearest-neighbor-like generalized (NNge) | x | x | x | x | x |  | [(Martin 1995)](https://paperpile.com/c/aQ1u2M/Ec2EW) |
|  | Ordinal Learning Method (OLM) | x | x | x | x | x |  | [(Ben-David 1992)](https://paperpile.com/c/aQ1u2M/0o0oO) |
|  | One Rule (OneR) | x | x | x | x | x |  | [(Ben-David 1992; Holte 1993)](https://paperpile.com/c/aQ1u2M/0o0oO+kDJAX) |
|  | PART | x | x | x | x | x |  | [(Frank and Witten 1998)](https://paperpile.com/c/aQ1u2M/KEBFn) |
|  | PRISM | x |  |  | x | x |  | [(Jiménez, Sánchez, and Juárez 2014; Cendrowska 1987)](https://paperpile.com/c/aQ1u2M/HA6tj+P5f2O) |
|  | RIpple-DOwn Rule (Ridor) | x | x | x | x | x |  | [(Compton and Jansen 1990)](https://paperpile.com/c/aQ1u2M/ZBQ8Q) |
|  | Rough Set | x | x | x | x | x |  | [(Bazan and Szczuka 2001)](https://paperpile.com/c/aQ1u2M/Kevc5) |
|  | Zero Rule (ZeroR) | x | x | x | x | x | x | [(Holmes, Donkin, and Witten 1994)](https://paperpile.com/c/aQ1u2M/wjH1R) |
| Trees | Alternating Decision Tree (ADTree) | x | x | x |  | x |  | [(Bazan and Szczuka 2001; Yoav Freund 1999)](https://paperpile.com/c/aQ1u2M/Kevc5+7iR5w) |
|  | Alternating Model Tree | x | x | x |  |  | x | [(Frank, Mayo, and Kramer 2015)](https://paperpile.com/c/aQ1u2M/6Kjw1) |
|  | Best-First Decision Tree (BFTree) | x | x | x | x | x |  | [(Shi 2007)](https://paperpile.com/c/aQ1u2M/om4aA) |
|  | Credal Decision Tree (CDT) | x | x | x | x | x | x | [(Mantas and Abellán 2014)](https://paperpile.com/c/aQ1u2M/vpsuk) |
|  | Decision Stump | x | x | x | x | x | x | [(Fürnkranz 2016)](https://paperpile.com/c/aQ1u2M/3VqDy) |
|  | ExtraTree |  | x |  | x | x | x | [(Geurts, Ernst, and Wehenkel 2006)](https://paperpile.com/c/aQ1u2M/F8B3K) |
|  | Functional Trees (FT) | x | x | x | x | x |  | [(Landwehr, Hall, and Frank 2005)](https://paperpile.com/c/aQ1u2M/d1IP0) |
|  | Hoeffding Tree | x | x | x | x | x |  | [(Hulten, Spencer, and Domingos 2001)](https://paperpile.com/c/aQ1u2M/T8Zqy) |
|  | ID3 | x |  |  | x | x |  | [(Landwehr, Hall, and Frank 2005; J. R. Quinlan 1986)](https://paperpile.com/c/aQ1u2M/d1IP0+NH5fo) |
|  | C4.5 Tree (J48) | x | x | x | x | x |  | [(John Ross Quinlan 1993)](https://paperpile.com/c/aQ1u2M/yGKLd) |
|  | C4.5 Consolidated Tree (J48 Consolidated) | x | x | x | x | x |  | [(Pérez et al. 2007; Ibarguren et al. 2015)](https://paperpile.com/c/aQ1u2M/s43NR+bhSu2) |
|  | LogitBoost Alternating Decision Tree (LADTree) | x | x | x | x | x |  | [(Holmes et al. 2002)](https://paperpile.com/c/aQ1u2M/vUAqe) |
|  | Logistic Model Trees (LMT) | x | x | x | x | x |  | [(Landwehr, Hall, and Frank 2005; Sumner, Frank, and Hall 2005)](https://paperpile.com/c/aQ1u2M/d1IP0+wFFqo) |
|  | M5 Model Trees | x | x | x |  |  | x | [(J. R. Quinlan 1992; Wang, Witten, and University of Waikato. Department of Computer Science 1996)](https://paperpile.com/c/aQ1u2M/T0eTR+HiRLZ) |
|  | Naive Bayes Tree (NBTree) | x | x | x | x | x |  | [(Kohavi 1996)](https://paperpile.com/c/aQ1u2M/tKYz2) |
|  | Random Forest | x | x | x | x | x | x | [(Breiman 2001)](https://paperpile.com/c/aQ1u2M/aAAy1) |
|  | Random Tree | x | x | x | x | x | x | [(Holmes, Donkin, and Witten 1994)](https://paperpile.com/c/aQ1u2M/wjH1R) |
|  | Fast Decision Tree Learner (REPTree) | x | x | x | x | x | x | [(C 2015)](https://paperpile.com/c/aQ1u2M/0CSbP) |
|  | SimpleCart | x | x | x | x | x |  | [(Breiman et al. 1984)](https://paperpile.com/c/aQ1u2M/PZfvs) |

**Table S2: Supported criteria (i.e. metrics) to optimize feature search with their abbreviation (Abbv.), optimization procedure (maximization (Max.) or minimization (Min.)), category (Cat.) of classifier (classification (Class.) or regression (Reg.)), formula and reference. Criterions are computed using the number of false positives (FP, i.e. type I error), true negatives (TN, i.e. type II error), false negatives (FN) and true positives (TP).**

| **Criterion** | **Abbv.** | **Opt.** | **Cat.** | **Formula** | **Ref.** |
| --- | --- | --- | --- | --- | --- |
| Accuracy | ACC | Max. | Class. | 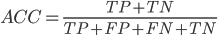 | [(Leclercq, Diallo, and Blanchette 2013)](https://paperpile.com/c/aQ1u2M/bIVyO) |
| Balanced Error Rate | BER | Min. | Class. | 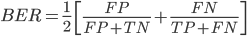 | [(Chen and Lin, n.d.; McAuley and Leskovec 2012)](https://paperpile.com/c/aQ1u2M/CiH4h+3Dw9m) |
| F1 score (also F-score or F-measure) | F-score | Max. | Class. | 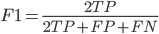 | [(Witten et al. 2016)](https://paperpile.com/c/aQ1u2M/g4ZoG) |
| Kappa | Kappa | Max. | Class. | 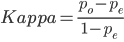with 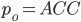and 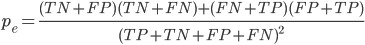 | [(Landis, Richard Landis, and Koch 1977)](https://paperpile.com/c/aQ1u2M/UCZWb) |
| Matthew’s correlation coefficient | MCC | Max. | Class. | 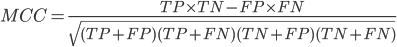 | [(Matthews 1975)](https://paperpile.com/c/aQ1u2M/r7YMw) |
| Sensitivity (or True Positive Rate) | TPR | Max. | Class. | 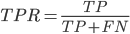 | [(Leclercq, Diallo, and Blanchette 2013)](https://paperpile.com/c/aQ1u2M/bIVyO) |
| Specificity (or recall, True Negative Rate) | TNR | Max. | Class. | 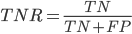 | [(Leclercq, Diallo, and Blanchette 2013)](https://paperpile.com/c/aQ1u2M/bIVyO) |
| Precision | Precision | Max. | Class. | 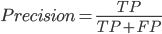 | [(Witten et al. 2016)](https://paperpile.com/c/aQ1u2M/g4ZoG) |
| Correlation coefficient (Pearson's) | CC | Max. | Reg. | 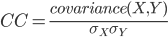where 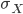is the standard deviation of 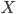 | [(“Typical Laws of Heredity 1” 1877)](https://paperpile.com/c/aQ1u2M/hPEUW) |
| False Discovery Rate | FDR | Min. | Reg. | 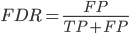 | [(Witten et al. 2016)](https://paperpile.com/c/aQ1u2M/g4ZoG) |
| Mean Absolute Error | MAE | Min. | Reg. | 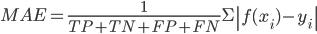  with 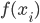the predicted value of the *i*-th example and 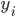 the true value | [(Willmott and Matsuura 2005)](https://paperpile.com/c/aQ1u2M/IX23j) |
| Relative absolute error | RAE | Min. | Reg. | 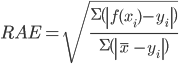 | [(Armstrong and Collopy 1993)](https://paperpile.com/c/aQ1u2M/u8M9t) |
| Root Mean Squared Error | RMSE | Min. | Reg. | 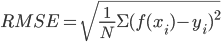 | [(Willmott and Matsuura 2005)](https://paperpile.com/c/aQ1u2M/IX23j) |
| Root Relative Squared Error | RRSE | Min. | Reg. | 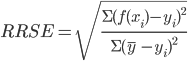 | [(S. Zhang, Caragea, and Ou 2011)](https://paperpile.com/c/aQ1u2M/7t1Pn) |
| Area Under Receiving Operating Characteristic curve | AUC | Max. | Class. | Calculated using the trapezoidal areas created between each receiving operating curve points. Equivalent to the Wilcoxon-Mann-Whitney statistic. | [(Davis and Goadrich 2006)](https://paperpile.com/c/aQ1u2M/HfShE) |
| Area Under the Precision Recall Curve | AUPRC | Max. | Class. | 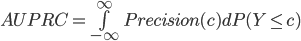  Where 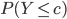the probability of positive examples inferior to given threshold *c* | [(Boyd, Eng, and David Page 2013)](https://paperpile.com/c/aQ1u2M/XT757) |

# **Supplementary results**

# **2.1. Datasets sources**

Datasets used in the study for benchmarking BioDiscML are available for download at:

- ColonCA. <http://eps.upo.es/bigs/datasets.html>
- Central Nervous System: <http://eps.upo.es/bigs/datasets.html>
- Dlbcl: <http://ico2s.org/datasets/microarray.html>
- Prostate-Singh: <http://ico2s.org/datasets/microarray.html>

# **2.2. BioDiscML VS MINT analysis**

See Datasets_results.xlsx containing all generated models.

**2.2.1. Genes signatures**

**Stem cell dataset short signature identified by MINT:**

*ENSG00000131914, ENSG00000154639, ENSG00000137871, ENSG00000129317, ENSG00000155008, ENSG00000095261, ENSG00000171466, ENSG00000214553, ENSG00000149531, ENSG00000102226, ENSG00000188372, ENSG00000243708, ENSG00000123562, ENSG00000220023, ENSG00000108479, ENSG00000109536, ENSG00000137806*

**Stem cell dataset short signature identified by BioDiscML:**

*ENSG00000046653, ENSG00000068079, ENSG00000069482, ENSG00000093010, ENSG00000115457, ENSG00000123975, ENSG00000130203, ENSG00000141682, ENSG00000147145, ENSG00000163191, ENSG00000163251, ENSG00000164045, ENSG00000166165, ENSG00000169231, ENSG00000173660, ENSG00000174442, ENSG00000178445, ENSG00000180543, ENSG00000187742*

**Stem cell dataset short signature WITH correlated features identified by BioDiscML:**

*ENSG00000002330, ENSG00000004779, ENSG00000046653, ENSG00000068079, ENSG00000069482, ENSG00000080561, ENSG00000082515, ENSG00000086475, ENSG00000087586, ENSG00000093010, ENSG00000101040, ENSG00000103275, ENSG00000104147, ENSG00000106066, ENSG00000106278, ENSG00000115457, ENSG00000116260, ENSG00000119782, ENSG00000119922, ENSG00000120992, ENSG00000121022, ENSG00000121570, ENSG00000123975, ENSG00000124570, ENSG00000124802, ENSG00000125375, ENSG00000125843, ENSG00000127184, ENSG00000128340, ENSG00000129460, ENSG00000130203, ENSG00000131914, ENSG00000132780, ENSG00000136867, ENSG00000136943, ENSG00000138363, ENSG00000140025, ENSG00000141682, ENSG00000144579, ENSG00000146757, ENSG00000147145, ENSG00000147586, ENSG00000151150, ENSG00000151806, ENSG00000152413, ENSG00000154639, ENSG00000162972, ENSG00000163191, ENSG00000163251, ENSG00000164045, ENSG00000166165, ENSG00000166173, ENSG00000167642, ENSG00000169020, ENSG00000169231, ENSG00000170312, ENSG00000170522, ENSG00000173207, ENSG00000173262, ENSG00000173660, ENSG00000174442, ENSG00000178445, ENSG00000180543, ENSG00000187742, ENSG00000196290, ENSG00000198873, ENSG00000204531, ENSG00000221869, ENSG00000225976, ENSG00000233954, ENSG00000241837*

**2.2.2. Ontology analysis**


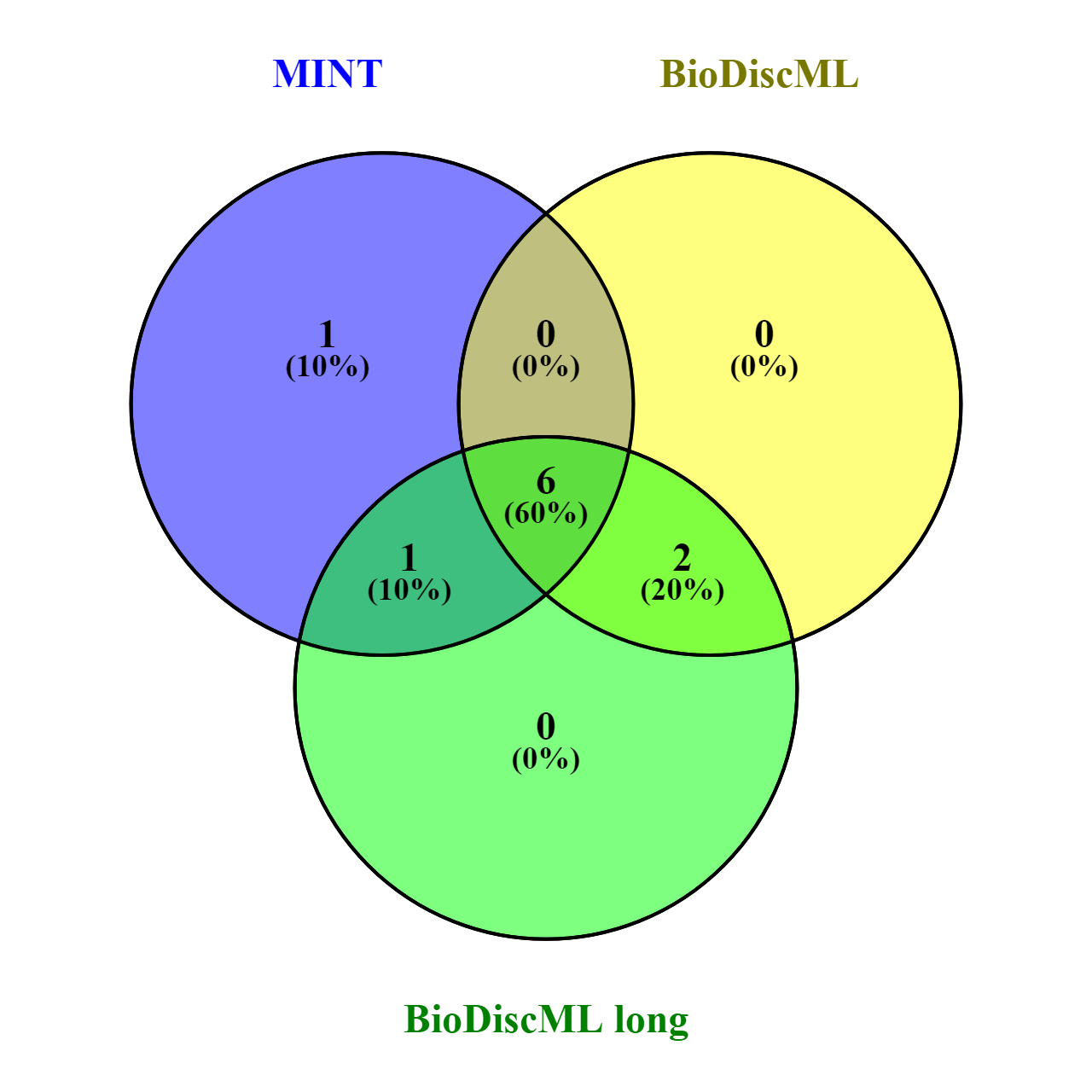


**Supplementary Figure S2: VENN DIAGRAM figure of Biological processes of signatures obtained by Mint, BiodiscML and BiodiscML with correlated genes. Ontologies were retrieved using PANTHER tool** [**(Mi et al. 2017)**](https://paperpile.com/c/aQ1u2M/sCUiN)**.**

**Venn diagram (Supplementary Figure S2) content:**

Mint (8 Biological processes):

reproduction (GO:0000003)

immune system process (GO:0002376)

cellular process (GO:0009987)

multicellular organismal process (GO:0032501)

metabolic process (GO:0008152)

biological regulation (GO:0065007)

cellular component organization or biogenesis (GO:0071840)

localization (GO:0051179)

BioDiscML (8 Biological processes):

response to stimulus (GO:0050896)

developmental process (GO:0032502)

cellular process (GO:0009987)

multicellular organismal process (GO:0032501)

metabolic process (GO:0008152)

biological regulation (GO:0065007)

cellular component organization or biogenesis (GO:0071840)

localization (GO:0051179)

BioDiscML long (with correlated genes) (9 Biological processes):

cellular component organization or biogenesis (GO:0071840)

cellular process (GO:0009987)

localization (GO:0051179)

biological regulation (GO:0065007)

response to stimulus (GO:0050896)

developmental process (GO:0032502)

multicellular organismal process (GO:0032501)

metabolic process (GO:0008152)

immune system process (GO:0002376)

1 element included exclusively in "MINT":

reproduction (GO:0000003)

2 common elements in "BioDiscML " and "BioDiscML long":

response to stimulus (GO:0050896)

developmental process (GO:0032502)

1 common element in "MINT" and "BioDiscML long":

immune system process (GO:0002376)

6 common elements in "MINT", "BioDiscML " and "BioDiscML long":

cellular process (GO:0009987)

multicellular organismal process (GO:0032501)

metabolic process (GO:0008152)

biological regulation (GO:0065007)

cellular component organization or biogenesis (GO:0071840)

# localization (GO:0051179)**2.3. BioDiscML VS RGIFE datasets analysis**

Affymetrix aliases were converted using GProfiler [(Reimand et al., 2016)](https://paperpile.com/c/aQ1u2M/iMiZ) with AFFY_HUGENEFLAFFX namespace.

**2.2.1. CNS signature**

**HoeffdingTree model**

| **initial_alias** | **converted_alias** | **name** |
| --- | --- | --- |
| AFFX-CREX-3_ST | None | None |
| HG2417-HT2513_AT | None | None |
| L13923_AT | ENSG00000166147 | FBN1 |
| M18728_AT | ENSG00000086548 | CEACAM6 |
| M55593_AT | ENSG00000087245 | MMP2 |
| M63962_RNA1_AT | ENSG00000105675 | ATP4A |
| U37673_AT | ENSG00000103723 | AP3B2 |
| U41737_AT | None | None |
| U43747_S_AT | ENSG00000165060 | FXN |
| X54938_AT | ENSG00000137825 | ITPKA |
| X80230_AT | ENSG00000136807 | CDK9 |

**Bayes A2DE model**

| **initial_alias** | **converted_alias** | **name** |
| --- | --- | --- |
| AF001787_S_AT | ENSG00000175564 | UCP3 |
| AFFX-CREX-3_ST | None | None |
| D86479_AT | ENSG00000106624 | AEBP1 |
| D86974_AT | ENSG00000169203 | NPIPB12 |
| D86974_AT | ENSG00000169246 | NPIPB3 |
| D86974_AT | ENSG00000185864 | NPIPB4 |
| D86974_AT | ENSG00000198064 | NPIPB13 |
| D86974_AT | ENSG00000243716 | NPIPB5 |
| D86974_AT | ENSG00000254206 | NPIPB11 |
| M18728_AT | ENSG00000086548 | CEACAM6 |
| M55593_AT | ENSG00000087245 | MMP2 |
| M60748_AT | ENSG00000168298 | HIST1H1E |
| M63962_RNA1_AT | ENSG00000105675 | ATP4A |
| M96739_AT | ENSG00000171786 | NHLH1 |
| U28963_AT | ENSG00000132522 | GPS2 |
| U28963_AT | ENSG00000261915 | AC026954.2 |
| U29943_S_AT | ENSG00000107105 | ELAVL2 |
| X54938_AT | ENSG00000137825 | ITPKA |

**Genes overlapping between both signatures (5):**

AFFX-CreX-3_st

M18728_at

M55593_at

M63962_rna1_at

X54938_at

**2.2.1. DLBCL signature**

**NaiveBayes model**

| initial_alias | converted_alias | name |
| --- | --- | --- |
| D26361_AT | ENSG00000118193 | KIF14 |
| M12963_S_AT | ENSG00000187758 | ADH1A |
| M12963_S_AT | ENSG00000196616 | ADH1B |
| M12963_S_AT | ENSG00000248144 | ADH1C |
| M23323_S_AT | ENSG00000198851 | CD3E |
| M31724_AT | ENSG00000196396 | PTPN1 |
| M63835_AT | ENSG00000150337 | FCGR1A |
| M63835_AT | ENSG00000198019 | FCGR1B |
| M63835_AT | ENSG00000265531 | FCGR1CP |
| U63743_AT | ENSG00000142945 | KIF2C |

**A1DE model**

| initial_alias | converted_alias | name |
| --- | --- | --- |
| D25218_AT | ENSG00000179041 | RRS1 |
| D80008_AT | ENSG00000101003 | GINS1 |
| HG4074-HT4344_AT | None | None |
| U77735_AT | ENSG00000102096 | PIM2 |
| X69090_AT | ENSG00000101605 | MYOM1 |
| Z11793_AT | ENSG00000250722 | SELENOP |

**Genes overlapping between both signatures (0):**

None

**2.2.1. Prostate cancer signature**

**VFI model**

| initial_alias | converted_alias | name |
| --- | --- | --- |
| 1944_F_AT | None | None |
| 216_AT | ENSG00000107317 | PTGDS |
| 31427_AT | None | None |
| 31687_F_AT | ENSG00000223609 | HBD |
| 31687_F_AT | ENSG00000244734 | HBB |
| 32269_AT | None | None |
| 34310_AT | ENSG00000198931 | APRT |
| 36629_AT | ENSG00000157514 | TSC22D3 |
| 36635_AT | ENSG00000058063 | ATP11B |
| 38407_R_AT | ENSG00000107317 | PTGDS |
| 38407_R_AT | ENSG00000284341 | AL807752.7 |
| 38779_R_AT | ENSG00000143321 | HDGF |
| 40783_S_AT | ENSG00000183506 | PI4KAP2 |
| 40783_S_AT | ENSG00000241973 | PI4KA |
| 40783_S_AT | ENSG00000274602 | PI4KAP1 |
| 41430_AT | ENSG00000091157 | WDR7 |

**Naïve Bayes Model**

| initial_alias | converted_alias | name |
| --- | --- | --- |
| 1184_AT | ENSG00000100911 | PSME2 |
| 1184_AT | ENSG00000238000 | AC116347.1 |
| 1944_F_AT | None | None |
| 38779_R_AT | ENSG00000143321 | HDGF |
| 39220_AT | ENSG00000149021 | SCGB1A1 |
| 40282_S_AT | ENSG00000197766 | CFD |
| 40304_AT | ENSG00000151914 | DST |
| 41430_AT | ENSG00000091157 | WDR7 |
| 41817_G_AT | ENSG00000100065 | CARD10 |
| 424_S_AT | ENSG00000077782 | FGFR1 |
| 914_G_AT | ENSG00000157554 | ERG |

**Genes overlapping between both signatures (0):**

None

1. **Supplementary References**

[Aha, D. W., Kibler, D., and Albert, M. K. (1991). Instance-Based Learning Algorithms. *Mach. Learn.* 6, 37–66.](http://paperpile.com/b/aQ1u2M/XA77G)

[Andrew McCallum, K. N. (1998). A comparison of event models for Naive Bayes text classification. in *IN AAAI-98 WORKSHOP ON LEARNING FOR TEXT CATEGORIZATION* Available at:](http://paperpile.com/b/aQ1u2M/TY5s0) <http://citeseerx.ist.psu.edu/viewdoc/summary?doi=10.1.1.65.9324> [[Accessed June 15, 2018].](http://paperpile.com/b/aQ1u2M/TY5s0)

[Armstrong, J. S., and Collopy, F. (1993). Error measures for generalizing about forecasting methods: Empirical comparisons. *Long Range Plann.* 26, 150.](http://paperpile.com/b/aQ1u2M/u8M9t)

[Atkeson, C. G., Moore, A. W., and Schaal, S. (1997). Locally Weighted Learning. *Artificial Intelligence Review* 11, 11–73.](http://paperpile.com/b/aQ1u2M/EcPUa)

[Baum, L. E., and Petrie, T. (1966). Statistical Inference for Probabilistic Functions of Finite State Markov Chains. *Ann. Math. Stat.* 37, 1554–1563.](http://paperpile.com/b/aQ1u2M/NHlf8)

[Bazan, J. G., and Szczuka, M. (2001). “RSES and RSESlib - A Collection of Tools for Rough Set Computations,” in *Lecture Notes in Computer Science*, 106–113.](http://paperpile.com/b/aQ1u2M/Kevc5)

[Ben-David, A. (1992). Automatic Generation of Symbolic Multiattribute Ordinal Knowledge-Based DSSs: Methodology and Applications. *Decision Sciences* 23, 1357–1372.](http://paperpile.com/b/aQ1u2M/0o0oO)

[Blei, D. M., Ng, A. Y., and Jordan, M. I. (2003). Latent Dirichlet Allocation. *J. Mach. Learn. Res.* 3, 993–1022.](http://paperpile.com/b/aQ1u2M/yJ3vL)

[Bouckaert, R. R. (2004). Bayesian network classifiers in Weka. Available at:](http://paperpile.com/b/aQ1u2M/6xwxZ) <https://researchcommons.waikato.ac.nz/handle/10289/85> [[Accessed June 18, 2018].](http://paperpile.com/b/aQ1u2M/6xwxZ)

[Boyd, K., Eng, K. H., and David Page, C. (2013). “Area under the Precision-Recall Curve: Point Estimates and Confidence Intervals,” in *Lecture Notes in Computer Science*, 451–466.](http://paperpile.com/b/aQ1u2M/XT757)

[Breiman, L. (2001). Random Forests. *Mach. Learn.* 45, 5–32.](http://paperpile.com/b/aQ1u2M/aAAy1)

[Breiman, L., Friedman, J., Stone, C. J., and Olshen, R. A. (1984). *Classification and Regression Trees*. Taylor & Francis.](http://paperpile.com/b/aQ1u2M/PZfvs)

[Cao-Van, K. (2003). Supervised ranking, from semantics to algorithms. doi:](http://paperpile.com/b/aQ1u2M/ZsbQC)[10.13140/RG.2.1.3756.4967](http://dx.doi.org/10.13140/RG.2.1.3756.4967)[.](http://paperpile.com/b/aQ1u2M/ZsbQC)

[Cendrowska, J. (1987). PRISM: An algorithm for inducing modular rules. *Int. J. Man. Mach. Stud.* 27, 349–370.](http://paperpile.com/b/aQ1u2M/P5f2O)

[Cessie, S. L., Le Cessie, S., and Van Houwelingen, J. C. (1992). Ridge Estimators in Logistic Regression. *Appl. Stat.* 41, 191.](http://paperpile.com/b/aQ1u2M/2N4es)

[Chang, C.-C., and Lin, C.-J. (2011). LIBSVM: A library for support vector machines. *ACM Transactions on Intelligent Systems and Technology (TIST)* 2, 27.](http://paperpile.com/b/aQ1u2M/XxwnM)

[Chen, Y.-W., and Lin, C.-J. “Combining SVMs with Various Feature Selection Strategies,” in *Studies in Fuzziness and Soft Computing*, 315–324.](http://paperpile.com/b/aQ1u2M/CiH4h)

[C, L. D. (2015). Proficiency Comparison Ofladtree And Reptree Classifiers For Credit Risk Forecast. *International Journal on Computational Science & Applications* 5, 39–50.](http://paperpile.com/b/aQ1u2M/0CSbP)

[Cleary, J. G., and Trigg, L. E. (1995). K*: an instance-based learner using and entropic distance measure. in *Proceedings of the Twelfth International Conference on International Conference on Machine Learning* (Morgan Kaufmann Publishers Inc.), 108–114.](http://paperpile.com/b/aQ1u2M/drSQh)

[Cohen, W. W. (1995). “Fast Effective Rule Induction,” in *Machine Learning Proceedings 1995*, 115–123.](http://paperpile.com/b/aQ1u2M/eVQVV)

[Compton, P., and Jansen, R. (1990). “Knowledge in context: A strategy for expert system maintenance,” in *Lecture Notes in Computer Science*, 292–306.](http://paperpile.com/b/aQ1u2M/ZBQ8Q)

[Cover, T. M. (1965). Geometrical and Statistical Properties of Systems of Linear Inequalities with Applications in Pattern Recognition. *IEEE Trans. Comput.* EC-14, 326–334.](http://paperpile.com/b/aQ1u2M/BmX2S)

[Cristianini, N. (2004). “Fisher Discriminant Analysis (Linear Discriminant Analysis),” in *Dictionary of Bioinformatics and Computational Biology*.](http://paperpile.com/b/aQ1u2M/PHOLF)

[Davis, J., and Goadrich, M. (2006). The relationship between Precision-Recall and ROC curves. in *Proceedings of the 23rd international conference on Machine learning - ICML ’06* doi:](http://paperpile.com/b/aQ1u2M/HfShE)[10.1145/1143844.1143874](http://dx.doi.org/10.1145/1143844.1143874)[.](http://paperpile.com/b/aQ1u2M/HfShE)

[Demiröz, G., and Altay Güvenir, H. (1997). “Classification by Voting Feature Intervals,” in *Lecture Notes in Computer Science*, 85–92.](http://paperpile.com/b/aQ1u2M/ihnLA)

[Duda, R. O., and Hart, P. E. (1973). *Pattern Classification and Scene Analysis*. Wiley.](http://paperpile.com/b/aQ1u2M/GyvM1)

[Fan, R.-E., Chang, K.-W., Hsieh, C.-J., Wang, X.-R., and Lin, C.-J. (2008). LIBLINEAR: A Library for Large Linear Classification. *J. Mach. Learn. Res.* 9, 1871–1874.](http://paperpile.com/b/aQ1u2M/cGPhj)

[Frank, E. (2014). Fully supervised training of Gaussian radial basis function networks in WEKA. 1–5.](http://paperpile.com/b/aQ1u2M/vEYff)

[Frank, E., Hall, M., and Pfahringer, B. (2002). Locally weighted naive bayes. in *Proceedings of the Nineteenth conference on Uncertainty in Artificial Intelligence* (Morgan Kaufmann Publishers Inc.), 249–256.](http://paperpile.com/b/aQ1u2M/rjTlq)

[Frank, E., Mayo, M., and Kramer, S. (2015). Alternating model trees. in *Proceedings of the 30th Annual ACM Symposium on Applied Computing* (ACM), 871–878.](http://paperpile.com/b/aQ1u2M/6Kjw1)

[Frank, E., and Witten, I. H. (1998). Generating Accurate Rule Sets Without Global Optimization. in *Proceedings of the Fifteenth International Conference on Machine Learning* (Morgan Kaufmann Publishers Inc.), 144–151.](http://paperpile.com/b/aQ1u2M/KEBFn)

[Freund, Y., and Schapire, R. E. (1998). Large margin classification using the perceptron algorithm. in *Proceedings of the eleventh annual conference on Computational learning theory* (ACM), 209–217.](http://paperpile.com/b/aQ1u2M/2hqLQ)

[Friedman, J., Hastie, T., and Tibshirani, R. (2010). Regularization Paths for Generalized Linear Models via Coordinate Descent. *J. Stat. Softw.* 33, 1–22.](http://paperpile.com/b/aQ1u2M/Jxnmr)

[Fürnkranz, J. (2016). “Decision Stump,” in *Encyclopedia of Machine Learning and Data Mining*, 1–1.](http://paperpile.com/b/aQ1u2M/3VqDy)

[Geurts, P., Ernst, D., and Wehenkel, L. (2006). Extremely randomized trees. *Mach. Learn.* 63, 3–42.](http://paperpile.com/b/aQ1u2M/F8B3K)

[Hall, M. A., and Frank, E. (2008). Combining Naive Bayes and Decision Tables. 318–319.](http://paperpile.com/b/aQ1u2M/veSV1)

[Hastie, T., and Tibshirani, R. (1998). Classification by pairwise coupling. in *Proceedings of the 1997 conference on Advances in neural information processing systems 10* (MIT Press), 507–513.](http://paperpile.com/b/aQ1u2M/iKQAc)

[Holmes, G., Donkin, A., and Witten, I. H. (1994). *WEKA: A Machine Learning Workbench*. University of Waikato. Department of Computer Science.](http://paperpile.com/b/aQ1u2M/wjH1R)

[Holmes, G., Hall, M., and Prank, E. (1999). “Generating Rule Sets from Model Trees,” in *Lecture Notes in Computer Science*, 1–12.](http://paperpile.com/b/aQ1u2M/daRbB)

[Holmes, G., Pfahringer, B., Kirkby, R., Frank, E., and Hall, M. (2002). Multiclass Alternating Decision Trees. in *Proceedings of the 13th European Conference on Machine Learning* (Springer-Verlag), 161–172.](http://paperpile.com/b/aQ1u2M/vUAqe)

[Holte, R. C. (1993). Very Simple Classification Rules Perform Well on Most Commonly Used Datasets. *Mach. Learn.* 11, 63–90.](http://paperpile.com/b/aQ1u2M/kDJAX)

[Hühn, J., and Hüllermeier, E. (2009). FURIA: an algorithm for unordered fuzzy rule induction. *Data Min. Knowl. Discov.* 19, 293–319.](http://paperpile.com/b/aQ1u2M/4THfq)

[Hulten, G., Spencer, L., and Domingos, P. (2001). Mining time-changing data streams. in *Proceedings of the seventh ACM SIGKDD international conference on Knowledge discovery and data mining - KDD ’01* doi:](http://paperpile.com/b/aQ1u2M/T8Zqy)[10.1145/502512.502529](http://dx.doi.org/10.1145/502512.502529)[.](http://paperpile.com/b/aQ1u2M/T8Zqy)

[Ibarguren, I., Pérez, J. M., Muguerza, J., Gurrutxaga, I., and Arbelaitz, O. (2015). Coverage-based resampling: Building robust consolidated decision trees. *Knowledge-Based Systems* 79, 51–67.](http://paperpile.com/b/aQ1u2M/bhSu2)

[Jiménez, F., Sánchez, G., and Juárez, J. M. (2014). Multi-objective evolutionary algorithms for fuzzy classification in survival prediction. *Artif. Intell. Med.* 60, 197–219.](http://paperpile.com/b/aQ1u2M/HA6tj)

[John, G. H., and Langley, P. (1995). Estimating continuous distributions in Bayesian classifiers. in *Proceedings of the Eleventh conference on Uncertainty in artificial intelligence* (Morgan Kaufmann Publishers Inc.), 338–345.](http://paperpile.com/b/aQ1u2M/HZl0K)

[Kaburlasos, V. G., Athanasiadis, I. N., and Mitkas, P. A. (2007). Fuzzy lattice reasoning (FLR) classifier and its application for ambient ozone estimation. *Int. J. Approx. Reason.* 45, 152–188.](http://paperpile.com/b/aQ1u2M/2GCz4)

[Keerthi, S. S., Shevade, S. K., Bhattacharyya, C., and Murthy, K. R. K. (2001). Improvements to Platt’s SMO Algorithm for SVM Classifier Design. *Neural Comput.* 13, 637–649.](http://paperpile.com/b/aQ1u2M/BNw2i)

[King, W. L., and Holt, J. R. (1970). Conjunctive and disjunctive rule learning as a function of age and forced verbalization. *J. Exp. Child Psychol.* 10, 100–111.](http://paperpile.com/b/aQ1u2M/FYpTo)

[Kohavi, R. (1996). Scaling up the accuracy of Naive-Bayes classifiers: a decision-tree hybrid. in *Proceedings of the Second International Conference on Knowledge Discovery and Data Mining* (AAAI Press), 202–207.](http://paperpile.com/b/aQ1u2M/tKYz2)

[Landis, J. R., Richard Landis, J., and Koch, G. G. (1977). The Measurement of Observer Agreement for Categorical Data. *Biometrics* 33, 159.](http://paperpile.com/b/aQ1u2M/UCZWb)

[Landwehr, N., Hall, M., and Frank, E. (2005). Logistic Model Trees. *Mach. Learn.* 59, 161–205.](http://paperpile.com/b/aQ1u2M/d1IP0)

[Leclercq, M., Diallo, A. B., and Blanchette, M. (2013). Computational prediction of the localization of microRNAs within their pre-miRNA. *Nucleic Acids Res.* 41, 7200–7211.](http://paperpile.com/b/aQ1u2M/bIVyO)

[Lievens, S., De Baets, B., and Cao-Van, K. (2008). A probabilistic framework for the design of instance-based supervised ranking algorithms in an ordinal setting. *Ann. Oper. Res.* 163, 115–142.](http://paperpile.com/b/aQ1u2M/Pnfqp)

[Littlestone, N. (1988). Learning Quickly When Irrelevant Attributes Abound: A New Linear-Threshold Algorithm. *Mach. Learn.* 2, 285–318.](http://paperpile.com/b/aQ1u2M/4HkbH)

[MacKay, D. J. C., and Mac, D. J. (2003). *Information Theory, Inference and Learning Algorithms*. Cambridge University Press.](http://paperpile.com/b/aQ1u2M/5lbEC)

[Mantas, C. J., and Abellán, J. (2014). Credal Decision Trees to Classify Noisy Data Sets. in *Hybrid Artificial Intelligence Systems* (Springer, Cham), 689–696.](http://paperpile.com/b/aQ1u2M/vpsuk)

[Martin, B. (1995). Instance-based learning: nearest neighbour with generalisation. Available at:](http://paperpile.com/b/aQ1u2M/Ec2EW) <https://researchcommons.waikato.ac.nz/handle/10289/1095> [[Accessed June 18, 2018].](http://paperpile.com/b/aQ1u2M/Ec2EW)

[Matthews, B. W. (1975). Comparison of the predicted and observed secondary structure of T4 phage lysozyme. *Biochim. Biophys. Acta* 405, 442–451.](http://paperpile.com/b/aQ1u2M/r7YMw)

[McAuley, J., and Leskovec, J. (2012). “Image Labeling on a Network: Using Social-Network Metadata for Image Classification,” in *Lecture Notes in Computer Science*, 828–841.](http://paperpile.com/b/aQ1u2M/3Dw9m)

[Mi, H., Huang, X., Muruganujan, A., Tang, H., Mills, C., Kang, D., et al. (2017). PANTHER version 11: expanded annotation data from Gene Ontology and Reactome pathways, and data analysis tool enhancements. *Nucleic Acids Res.* 45, D183–D189.](http://paperpile.com/b/aQ1u2M/sCUiN)

[Pérez, J. M., Muguerza, J., Arbelaitz, O., Gurrutxaga, I., and Martín, J. I. (2007). Combining multiple class distribution modified subsamples in a single tree. *Pattern Recognit. Lett.* 28, 414–422.](http://paperpile.com/b/aQ1u2M/s43NR)

[Platt, J. C. (1999). Fast training of support vector machines using sequential minimal optimization. in *Advances in kernel methods* (MIT Press), 185–208.](http://paperpile.com/b/aQ1u2M/qfNy5)

[Quinlan, J. R. (1986). Induction of Decision Trees. *Mach. Learn.* 1, 81–106.](http://paperpile.com/b/aQ1u2M/NH5fo)

[Quinlan, J. R. (1992). Learning With Continuous Classes. *5th Australian Joint Conference on Artificial Intelligence*. Available at:](http://paperpile.com/b/aQ1u2M/T0eTR) <http://citeseerx.ist.psu.edu/viewdoc/summary?doi=10.1.1.34.885> [[Accessed June 18, 2018].](http://paperpile.com/b/aQ1u2M/T0eTR)

[Quinlan, J. R. (1993). *C4.5: Programs for Machine Learning*. Morgan Kaufmann.](http://paperpile.com/b/aQ1u2M/yGKLd)

[Reimand, J., Arak, T., Adler, P., Kolberg, L., Reisberg, S., Peterson, H., et al. (2016). g:Profiler-a web server for functional interpretation of gene lists (2016 update). *Nucleic Acids Res.* 44, W83–9.](http://paperpile.com/b/aQ1u2M/iMiZ)

[Rennie, J. D. M., Shih, L., Teevan, J., and Karger, D. R. (2003). Tackling the poor assumptions of naive bayes text classifiers. in *Proceedings of the Twentieth International Conference on International Conference on Machine Learning* (AAAI Press), 616–623.](http://paperpile.com/b/aQ1u2M/CnN8P)

[Rosenblatt, F. (1961). PRINCIPLES OF NEURODYNAMICS. PERCEPTRONS AND THE THEORY OF BRAIN MECHANISMS. Spartan Books doi:](http://paperpile.com/b/aQ1u2M/nmIJH)[10.21236/ad0256582](http://dx.doi.org/10.21236/ad0256582)[.](http://paperpile.com/b/aQ1u2M/nmIJH)

[Rousseeuw, P. J., and Leroy, A. M. (1987). *Robust Regression and Outlier Detection*. Wiley.](http://paperpile.com/b/aQ1u2M/F7kLe)

[Shalev-Shwartz, S., and Ben-David, S. (2014). “Stochastic Gradient Descent,” in *Understanding Machine Learning*, 150–166.](http://paperpile.com/b/aQ1u2M/kqYjs)

[Shalev-Shwartz, S., Singer, Y., and Srebro, N. (2007). Pegasos: Primal Estimated sub-GrAdient SOlver for SVM. in *Proceedings of the 24th international conference on Machine learning* (ACM), 807–814.](http://paperpile.com/b/aQ1u2M/CjJbU)

[Shevade, S. K., Keerthi, S. S., Bhattacharyya, C., and Murthy, K. K. (2000). Improvements to the SMO algorithm for SVM regression. *IEEE Trans. Neural Netw.* 11, 1188–1193.](http://paperpile.com/b/aQ1u2M/Gw1MN)

[Shi, H. (2007). Best-first Decision Tree Learning. Available at:](http://paperpile.com/b/aQ1u2M/om4aA) <https://researchcommons.waikato.ac.nz/handle/10289/2317> [[Accessed June 18, 2018].](http://paperpile.com/b/aQ1u2M/om4aA)

[Simone, V., Marino, M., and Toraldo, G. “Isotonic Regression Problems,” in *Encyclopedia of Optimization*, 1176–1179.](http://paperpile.com/b/aQ1u2M/eeOsI)

[Skousen, R. (1989). *Analogical Modeling of Language*. Springer Science & Business Media.](http://paperpile.com/b/aQ1u2M/2XB8z)

[Smola, A. J., and Schölkopf, B. (2004). A tutorial on support vector regression. *Stat. Comput.* 14, 199–222.](http://paperpile.com/b/aQ1u2M/Az3yc)

[Stefanowski, J. “On Combined Classifiers, Rule Induction and Rough Sets,” in *Lecture Notes in Computer Science*, 329–350.](http://paperpile.com/b/aQ1u2M/GA2aP)

[Sumner, M., Frank, E., and Hall, M. (2005). Speeding up logistic model tree induction. in *Proceedings of the 9th European conference on Principles and Practice of Knowledge Discovery in Databases* (Springer-Verlag), 675–683.](http://paperpile.com/b/aQ1u2M/wFFqo)

[Theodoridis, S. (2015). “Stochastic Gradient Descent,” in *Machine Learning*, 161–231.](http://paperpile.com/b/aQ1u2M/qkqqR)

[Typical Laws of Heredity 1 (1877). *Nature* 15, 492–495.](http://paperpile.com/b/aQ1u2M/hPEUW)

[Veloso, A., Jr., W., and Zaki, M. (2006). Lazy Associative Classification. in *Sixth International Conference on Data Mining (ICDM’06)* doi:](http://paperpile.com/b/aQ1u2M/CO0vM)[10.1109/icdm.2006.96](http://dx.doi.org/10.1109/icdm.2006.96)[.](http://paperpile.com/b/aQ1u2M/CO0vM)

[von Eye, A., and Schuster, C. (1998). “Simple Linear Regression,” in *Regression Analysis for Social Sciences*, 7–41.](http://paperpile.com/b/aQ1u2M/CUuMW)

[Wang, Y., Witten, I. H., and University of Waikato. Department of Computer Science (1996). *Induction of Model Trees for Predicting Continuous Classes*. Poster papers of the 9th European Conference on Machine Learning.](http://paperpile.com/b/aQ1u2M/HiRLZ)

[Webb, G. I., Boughton, J. R., Zheng, F., Ting, K. M., and Salem, H. (2011). Learning by extrapolation from marginal to full-multivariate probability distributions: decreasingly naive Bayesian classification. *Mach. Learn.* 86, 233–272.](http://paperpile.com/b/aQ1u2M/ytP5X)

[Wilkinson, L., Anand, A., and Tuan, D. N. (2011). CHIRP: a new classifier based on composite hypercubes on iterated random projections. in *Proceedings of the 17th ACM SIGKDD international conference on Knowledge discovery and data mining* (ACM), 6–14.](http://paperpile.com/b/aQ1u2M/uUwDr)

[Willmott, C. J., and Matsuura, K. (2005). Advantages of the mean absolute error (MAE) over the root mean square error (RMSE) in assessing average model performance. *Clim. Res.* 30, 79–82.](http://paperpile.com/b/aQ1u2M/IX23j)

[Witten, I. H., Frank, E., Hall, M. A., and Pal, C. J. (2016). *Data Mining: Practical Machine Learning Tools and Techniques*. Morgan Kaufmann.](http://paperpile.com/b/aQ1u2M/g4ZoG)

[Wojna, A., and Kowalski, L. (2005). Rseslib: Programmer’s Guide. Available at:](http://paperpile.com/b/aQ1u2M/WLEmK) [http://rsproject. mimuw. edu. pl](about:blank)[.](http://paperpile.com/b/aQ1u2M/WLEmK)

[Yan, X. (2009). *Linear Regression Analysis: Theory and Computing*. World Scientific.](http://paperpile.com/b/aQ1u2M/EvMoh)

[Yoav Freund, L. M. (1999). The Alternating Decision Tree Learning Algorithm. in *In Machine Learning: Proceedings of the Sixteenth International Conference* Available at:](http://paperpile.com/b/aQ1u2M/7iR5w) <http://citeseerx.ist.psu.edu/viewdoc/summary?doi=10.1.1.19.3673> [[Accessed June 18, 2018].](http://paperpile.com/b/aQ1u2M/7iR5w)

[Zhang, H., Jiang, L., and Su, J. (2005). Hidden naive Bayes. in *Proceedings of the 20th national conference on Artificial intelligence - Volume 2* (AAAI Press), 919–924.](http://paperpile.com/b/aQ1u2M/g8p2I)

[Zhang, S., Caragea, D., and Ou, X. (2011). “An Empirical Study on Using the National Vulnerability Database to Predict Software Vulnerabilities,” in *Lecture Notes in Computer Science*, 217–231.](http://paperpile.com/b/aQ1u2M/7t1Pn)

[Zheng, Z., and Webb, G. I. (2000). Lazy Learning of Bayesian Rules. *Mach. Learn.* 41, 53–84.](http://paperpile.com/b/aQ1u2M/KWcOK)[Ziqiang Wang, Wang, Z., and Sun, X. (2010). Document classification algorithm based on kernel logistic regression. in *2010 2nd International Conference on Industrial and Information Systems* doi:](http://paperpile.com/b/aQ1u2M/9JGZY)[10.1109/indusis.2010.5565909](http://dx.doi.org/10.1109/indusis.2010.5565909)[.](http://paperpile.com/b/aQ1u2M/9JGZY)
